# Supplementary material for: Metabolomic Profile of Açai (Euterpe oleracea Mart., Euterpe precatoria Mart.), Mirití (Mauritia flexuosa L.), and Cupuassu (Theobroma grandiflorum (Wild. ex Spreng.) Schum) from Colombian Amazon: Insights into Nutritional Composition and Ripening Dynamics
Source: Int J Mol Sci. 2025 Dec 30;27(1):410. doi: 10.3390/ijms27010410 (PMC12785605; doi:10.3390/ijms27010410)
Supplement: Supplementary file 1 [file ijms-27-00410-s001.zip › ijms-4047019-supplementary.pdf]

Supplementary Material

Content:

Table S1. Metabolomic profile *Mauritia flexuosa* (pulp samples).

Table S2. Metabolomic profile *Euterpe oleracea* and *Euterpe precatoria* (pulp samples).

Table S3. Metabolomic profile *Theobroma grandiflorum* (pulp and seeds).

Figure S1. Chromatograms of the *Mauritia flexuosa* ripe pulp extract obtained by a) LC-QTOF-MS(-), b) LC-QTOF-MS(+), and c) GC-QTOF-MS.

Figure S2. Chromatograms of the *Mauritia flexuosa* unripe pulp extract obtained by **a)** LC-QTOF-MS(-), **b)** LC-QTOF-MS(+), and **c)** GC-QTOF-MS.

Figure S3. Chromatograms of the *Euterpe oleracea* ripe pulp extract obtained by **a)** LC-QTOF-MS(-), **b)** LC-QTOF-MS(+), and **c)** GC-QTOF-MS.

Figure S4. Chromatograms of the *Euterpe oleracea* intermediate pulp extract obtained by **a)** LC-QTOF-MS(-), **b)** LC-QTOF-MS(+), and **c)** GC-QTOF-MS.

Figure S5. Chromatograms of the *Euterpe oleracea* unripe pulp extract obtained by **a)** LC-QTOF-MS(-), **b)** LC-QTOF-MS(+), and **c)** GC-QTOF-MS.

Figure S6. Chromatograms of the *Euterpe precatoria* ripe pulp extract obtained by **a)** LC-QTOF-MS(-), **b)** LC-QTOF-MS(+), and **c)** GC-QTOF-MS.

Figure S7. Chromatograms of the *Euterpe precatoria* unripe pulp extract obtained by **a)** LC-QTOF-MS(-), **b)** LC-QTOF-MS(+), and **c)** GC-QTOF-MS.

Figure S8. Chromatograms of the *Theobroma grandiflorum* ripe pulp extract obtained by **a)** LC-QTOF-MS(-), **b)** LC-QTOF-MS(+), and **c)** GC-QTOF-MS.

Figure S9. Chromatograms of the *Theobroma grandiflorum* unripe pulp extract obtained by **a)** LC-QTOF-MS(-), **b)** LC-QTOF-MS(+), and **c)** GC-QTOF-MS.

Figure S10. Chromatograms of the *Theobroma grandiflorum* ripe seed extract obtained by **a)** LC-QTOF-MS(-), **b)** LC-QTOF-MS(+), and **c)** GC-QTOF-MS.

Figure S11. Chromatograms of the *Theobroma grandiflorum* unripe seed extract obtained by **a)** LC-QTOF-MS(-), **b)** LC-QTOF-MS(+), and **c)** GC-QTOF-MS.

Table S1. Metabolomic profile *Mauritia flexuosa* (pulp samples).

Compound name	Molecular formula	Molecular weight (g/mol)	RT (min)	Mass error (ppm)	Adduct	Analytical platform	DET	Main fragment ions (MS/MS)	Identification level	*M. flexuosa ripe* (%RA)	*M. flexuosa unripe* (%RA)
Alcohols and polyols											
Anhydroglucitol	$C_6H_{12}O_5$	164.0685	16.30	-	-	GC-QTOF-MS	-	117.0730, 73.0464	2	0.11	-
Butanediol	$C_4H_{10}O_2$	90.0681	6.47	-	-	GC-QTOF-MS	-	152.0530, 117.0730, 73.0464	2	0.15	0.03
Myo-Inositol	$C_6H_{12}O_6$	180.0634	19.54	-	-	GC-QTOF-MS	-	305.1429, 217.1075, 191.0922, 147.0656, 73.0464	2	7.00	4.12
Shikimic Acid	$C_7H_{10}O_5$	174.0528	16.57	-	-	GC-QTOF-MS	-	93.0349, 73.0296	2	4.51	3.83
Triethylene Glycol	$C_6H_{14}O_4$	150.0892	13.00	-	-	GC-QTOF-MS	-	117.0726, 116.0648, 101.0415, 75.0259, 73.0466	2	0.06	0.09
Hydroxymethylcatechol	$C_7H_8O_3$	140.0473	2.74	4	$[M+H-H_2O]^+$	LC-QTOF-MS	ESI +	123.0551	2	0.33	0.32
Quinic acid	$C_7H_{12}O_6$	192.0634	8.10	3	$[M-H]^-$	LC-QTOF-MS	ESI -	191.0554, 127.0390, 85.0290	2	0.05	0.44
Arabitol	$C_5H_{12}O_5$	152.0685	15.49	-	-	GC-QTOF-MS	-	204.0996, 147.0656, 73.0469	2	0.31	0.19
Erythritol	$C_4H_{10}O_4$	122.0579	13.20	-	-	GC-QTOF-MS	-	217.1075, 147.0656, 73.0464	2	0.02	0.01
Galactinol	$C_{12}H_{22}O_{11}$	342.1162	26.39	-	-	GC-QTOF-MS	-	281.0522, 207.0339, 147.0667, 73.0475	2	0.14	-
Mannitol	$C_6H_{12}O_6$	180.0634	17.15	-	-	GC-QTOF-MS	-	103,0735, 73.0469	2	0.21	11.97
Sorbitol	$C_6H_{14}O_6$	182.0790	18.00	-	-	GC-QTOF-MS	-	319.1573, 205.1086, 147.0656, 73.0464	2	0.12	0.30
Aldehydes and derivatives											
Dihydroxybenzaldehyde	$C_7H_6O_3$	138.0317	5.35	3	$[M+H]^+$	LC-QTOF-MS	ESI +	91.0189, 65.0033, 41.0043	2	0.02	0.02
Dimethoxybenzaldehyde	$C_9H_{10}O_3$	166.0630	18.80	4	$[M+H]^+$	LC-QTOF-MS	ESI +	155.0730, 123.0470	2	0.20	0.09
Alkaloids											
Swainsonine	$C_8H_{15}NO_3$	173.1052	1.77	3	$[M+H-H_2O]^+$	LC-QTOF-MS	ESI +	174.1125	2	0.15	0.09
Amines											
Amino-phenylethanol	$C_8H_{11}NO$	137.0841	7.71		$[M+H-H_2O]^+$	LC-QTOF-MS	ESI +	120.113	2	0.99	1.45
Octopamine	$C_8H_{11}NO_2$	153.0790	2.74	3	$[M+H-H_2O]^+$	LC-QTOF-MS	ESI +	136.0868	2	0.56	0.63
Ethanolamine	C_2H_7NO	61.0528	9.92	-	-	GC-QTOF-MS	-	175.1134, 174.1125, 147.0652, 86.0418, 73.0466	2	0.12	0.08
Amino acids, peptides, and analogues											
Alanine	$C_3H_7NO_2$	89.0477	7.51	-	-	GC-QTOF-MS	-	147.0651, 116.0887, 75.0258, 73.0465, 59.0309	2	0.21	0.18
Aminobutyric Acid	$C_4H_9NO_2$	103.0633	13.38	-	-	GC-QTOF-MS	-	174.1129, 147.0656, 73.0469	2	0.40	0.66

Glutamic Acid	$C_5H_9NO_4$	147.0532	14.44	-	-	GC-QTOF-MS	-	246.1337, 156.0836, 147.0653, 128.0888, 73.0466	2	0.02	0.03
Leucine	$C_6H_{13}NO_2$	131.0946	9.99	-	-	GC-QTOF-MS	-	147.0668, 73.0471	2	0.07	0.11
Norvaline	$C_5H_{11}NO_2$	117.0790	9.20	-	-	GC-QTOF-MS	-	174.1203, 95.0366, 73.0469	2	0.07	0.07
Proline	$C_5H_9NO_2$	115.0633	10.35	-	-	GC-QTOF-MS	-	103.0207, 75.0258, 73.0466	2	0.07	0.03
Serine	$C_3H_7NO_3$	105.0426	11.24	-	-	GC-QTOF-MS	-	147.0656, 132.0837, 116.0532, 73.0464	2	0.02	0.03
Threonine	$C_4H_9NO_3$	119.0582	11.60	-	-	GC-QTOF-MS	-	142.1037, 130.0681, 73.0464	2	0.03	0.04
Betaine	$C_5H_{11}NO_2$	117.0790	1.18	7	$[M+H]^+$	LC-QTOF-MS	ESI +	118.0864, 59.0733, 58.0656	2	0.24	0.02
Isoleucylproline	$C_{11}H_{20}N_2O_3$	228.1474	3.45	5	$[M+H]^+$	LC-QTOF-MS	ESI +	-	3	0.30	0.13
Phenylalanine	$C_9H_{11}NO_2$	165.0790	4.60	4	$[M+H]^+$	LC-QTOF-MS	ESI +	166.0865, 120.0807, 103.0545, 79.0554	2	0.22	0.26
Tyrosine	$C_9H_{11}NO_3$	181.0739	2.74	3	$[M+H]^+$	LC-QTOF-MS	ESI +	136.0755, 123.0450, 119.0498, 95.0509, 91.0542	2	0.28	0.25
Acetyl-Leucine	$C_8H_{15}NO_3$	173.1052	5.24	3	$[M+HCOOH-H]^-$	LC-QTOF-MS	ESI -	-	3	0.27	0.08
Dipeptide (Phenylalanyl-Glycine)	$C_{11}H_{14}N_2O_3$	222.1004	6.91	3	$[M+H-H_2O]^+$	LC-QTOF-MS	ESI +	-	3	0.07	0.05
Dipeptide (Asparaginyl-Tryptophan)	$C_{15}H_{18}N_4O_4$	318.1328	5.05	0	$[M+Na]^+$	LC-QTOF-MS	ESI +	-	3	0.02	0.03
Benzoic acids and derivatives											
Benzoic Acid	$C_7H_6O_2$	122.0368	9.60	-	-	GC-QTOF-MS	-	179.0528, 77.0383, 73.0464	2	0.01	-
Gallic Acid	$C_7H_6O_5$	170.0215	18.10	-	-	GC-QTOF-MS	-	281.0660, 73.0468	2	0.04	0.04
Hydroxybenzoic Acid	$C_7H_6O_3$	138.0317	14.49	-	-	GC-QTOF-MS	-	267.0870, 223.0972, 193.0678	2	0.01	-
Vanillic acid	$C_8H_8O_4$	168.0423	5.45	2	$[M-H]^-$	LC-QTOF-MS	ESI -	167.0368, 152.0118, 108.0219	2	0.19	0.24
Carbohydrates and carbohydrate conjugates											
Allose	$C_6H_{12}O_6$	180.0634	17.14	-	-	GC-QTOF-MS	-	-	2	13.64	0.68
Arabinonic Acid	$C_5H_{10}O_6$	166.0477	16.12	-	-	GC-QTOF-MS	-	-	2	0.06	-
Arabinose	$C_5H_{10}O_5$	150.0528	14.74	-	-	GC-QTOF-MS	-	-	2	-	-
Digalacturonic Acid	$C_{12}H_{18}O_{13}$	370.0747	25.86	-	-	GC-QTOF-MS	-	-	2	0.30	0.29
Diglycerol	$C_6H_{14}O_5$	166.0841	15.74	-	-	GC-QTOF-MS	-	-	2	0.21	0.10
Fructose	$C_6H_{12}O_6$	180.0634	17.50	-	-	GC-QTOF-MS	-	307.1579, 217.1076, 103.0575, 73.0469	2	7.83	21.62
Fucose	$C_6H_{12}O_5$	164.0685	15.83	-	-	GC-QTOF-MS	-	137.9876, 113.9643	2	-	-
Galactosamine	$C_6H_{13}NO_5$	179.0794	17.15	-	-	GC-QTOF-MS	-	-	2	-	-
Galactose	$C_6H_{12}O_6$	180.0634	17.61	-	-	GC-QTOF-MS	-	319.1573, 205.1066, 147.0656, 73.0464	2	0.88	0.78

Galacturonic Acid	$C_6H_{10}O_7$	194.0427	18.15	-	-	GC-QTOF-MS	-	333.1374, 160.0788, 73.0469	2	28.15	22.34
Gentiobiose	$C_5H_6O_5$	146.0215	25.29	-	-	GC-QTOF-MS	-	361.1698, 204.0997, 147.0656, 73.0464	2	0.06	-
Gluconic Acid	$C_6H_{12}O_7$	196.0583	18.67	-	-	GC-QTOF-MS	-	307.1586, 217.1075, 147.0656, 73.0476	2	0.46	0.05
Glucose	$C_6H_{12}O_6$	180.0634	17.55	-	-	GC-QTOF-MS	-	-	2	0.21	0.26
Glucuronic Acid	$C_6H_{10}O_7$	194.0427	18.90	-	-	GC-QTOF-MS	-	364.1802, 307.1586, 217.1095, 147.0656, 103.0572, 73.0476	2	-	0.18
Glucuronic Acid Lactone	$C_6H_8O_6$	176.0321	16.92	-	-	GC-QTOF-MS	-	-	2	0.38	-
Glyceric Acid	$C_3H_6O_4$	106.0266	10.83	-	-	GC-QTOF-MS	-	189.0768, 147.0656, 73.0464	2	0.03	-
Glycerol	$C_3H_8O_3$	92.0473	10.04	-	-	GC-QTOF-MS	-	205.1082, 147.0668, 73.0471	2	2.85	1.03
Glycerol O-Galactopyranoside	$C_9H_{18}O_8$	254.1002	21.50	-	-	GC-QTOF-MS	-	-	2	0.14	-
Lyxose	$C_6H_{10}O_7$	194.0427	14.74	-	-	GC-QTOF-MS	-	-	2	0.01	-
Maltose	$C_{12}H_{22}O_{11}$	342.1162	24.71	-	-	GC-QTOF-MS	-	361.1685, 204.0996, 73.0469	2	-	0.03
Mucic Acid	$C_6H_{10}O_8$	210.0376	19.13	-	-	GC-QTOF-MS	-	333.1378, 292.1351, 217.1091, 147.0668, 73.0471	2	0.17	-
Psicose	$C_6H_{12}O_6$	180.0634	16.12	-	-	GC-QTOF-MS	-	-	2	-	0.05
Ribose	$C_5H_{10}O_5$	150.0528	15.02	-	-	GC-QTOF-MS	-	217.1075, 147.0656, 103.0572, 73.0464	2	0.53	0.06
Saccharic Acid (Glucaric Acid)	$C_6H_{10}O_8$	210.0376	18.34	-	-	GC-QTOF-MS	-	333.1372, 147.0656, 73.0469	2	8.25	5.83
Sorbose	$C_6H_{12}O_6$	180.0634	16.88	-	-	GC-QTOF-MS	-	-	2	0.08	-
Sucrose	$C_{12}H_{22}O_{11}$	342.1162	23.75	-	-	GC-QTOF-MS	-	-	2	0.05	0.06
Tagatose	$C_6H_{12}O_6$	180.0634	17.74	-	-	GC-QTOF-MS	-	-	2	0.10	0.08
Xylonic Acid	$C_5H_{10}O_6$	166.0477	14.64	-	-	GC-QTOF-MS	-	-	2	-	0.05
Carboxylic acids and derivatives											
Citric Acid	$C_6H_8O_7$	192.0270	16.75	-	-	GC-QTOF-MS	-	465.1608, 375.1107, 347.1167, 273.0992, 183.0480, 147.0673, 73.0476	2	1.46	1.07
Fumaric Acid	$C_4H_4O_4$	116.0110	10.95	-	-	GC-QTOF-MS	-	-	2	0.01	0.01
Succinic Acid	$C_4H_6O_4$	118.0266	10.52	-	-	GC-QTOF-MS	-	147.0656, 73.0464	2	0.21	0.20
Isonicotinic Acid	$C_6H_5NO_2$	123.0320	1.24	5	$[M+H]^+$	LC-QTOF-MS	ESI +	124.0394, 96.0433, 80.0495	2	0.33	0.28
Phenylacetic Acid	$C_8H_8O_2$	136.0524	2.74	3	$[M+H-H_2O]^+$	LC-QTOF-MS	ESI +	107.0493, 95.0486, 79.0541, 77.0382	2	0.21	0.21
Cyclohexenones											
Dehydroshikimic Acid	$C_7H_8O_5$	172.0372	16.37	-	-	GC-QTOF-MS	-	-	2	-	0.05
Fatty acids and derivatives											

Caprylic Acid	$C_8H_{16}O_2$	144.1150	9.76	-	-	GC-QTOF-MS	-	201.1302, 132.0837, 116.0526, 73.0468	2	0.07	0.12
Elaidic Acid	$C_{18}H_{34}O_2$	282.2559	20.35	-	-	GC-QTOF-MS	-	339.2744, 145.0695, 117.0381, 95.0856, 75.0267	2	2.06	-
Ethylhexanoic Acid	$C_8H_{16}O_2$	144.1150	7.41	-	-	GC-QTOF-MS	-	201.1312, 147.0656, 73.0464	2	0.08	0.14
Heptadecanoic Acid	$C_{17}H_{34}O_2$	270.2559	19.68	-	-	GC-QTOF-MS	-	-	2	0.01	0.01
Heptanoic Acid	$C_7H_{14}O_2$	130.0994	8.42	-	-	GC-QTOF-MS	-	147.0656, 117.0371, 75.0264	2	0.02	0.02
Heptanol	$C_7H_{16}O$	116.1201	6.22	-	-	GC-QTOF-MS	-	117.0740, 75.0260, 52.0308	2	0.06	-
Hexanoic Acid (Caproic Acid)	$C_6H_{12}O_2$	116.0837	7.00	-	-	GC-QTOF-MS	-	173.0990, 117.0367, 75.0261	2	0.02	0.02
Hydroxyisovalerate	$C_5H_{10}O_3$	118.0630	9.08	-	-	GC-QTOF-MS	-	171.0668, 147.0668, 131.0892, 117.0366, 95.0370, 75.0260	2	-	-
Lauric Acid	$C_{12}H_{24}O_2$	200.1776	14.70	-	-	GC-QTOF-MS	-	257.1930, 191.0916, 129.0369, 117.0367, 73.0468	2	-	-
Maltitol	$C_{12}H_{24}O_{11}$	344.1319	24.71	-	-	GC-QTOF-MS	-	-	2	0.20	-
Myristic Acid	$C_{14}H_{28}O_2$	228.2089	16.83	-	-	GC-QTOF-MS	-	285.2247, 129.0367, 117.0366, 75.0261	2	0.04	0.04
Octadecanol	$C_{18}H_{38}O$	270.2923	19.79	-	-	GC-QTOF-MS	-	327.3077, 97.1014, 75.0261, 57.0700	2	0.03	0.04
Oleic Acid	$C_{18}H_{34}O_2$	282.2559	20.34	-	-	GC-QTOF-MS	-	207.0323, 147.0656, 117.0356, 75.0252	2	-	1.83
Palmitic Acid	$C_{16}H_{32}O_2$	256.2402	18.77	-	-	GC-QTOF-MS	-	313.2577, 132.0683, 117.0371, 73.0464	2	1.93	2.16
Stearic Acid	$C_{18}H_{36}O_2$	284.2715	20.55	-	-	GC-QTOF-MS	-	341.2877, 145.0680, 117.0368, 73.0469	2	2.08	2.25
Octadecenamide (Oleamide)	$C_{18}H_{35}NO$	281.2719	18.53	4	$[M+H]^+$	LC-QTOF-MS	ESI +	-	3	0.29	0.16
HODE	$C_{18}H_{32}O_3$	296.2351	18.30	2	$[M+HCOOH-H]^-$	LC-QTOF-MS	ESI -	-	3	0.01	0.01
LPI 16:0	$C_{25}H_{49}O_{12}P$	572.2962	18.87	2	$[M-H]^-$	LC-QTOF-MS	ESI -	571.2884	2	0.04	-
Flavonoids											
2',6'-Dihydroxy-4'-methoxydihydrochalcone	$C_{16}H_{16}O_4$	272.1049	17.93	2	$[M+H]^+$	LC-QTOF-MS	ESI +	273.0784, 153.0159, 147.1191	2	0.47	-
Procyanidin B2	$C_{30}H_{26}O_{12}$	578.1424	7.34	2	$[M+H]^+$	LC-QTOF-MS	ESI +	541.1018, 425.0862, 407.0776, 289.0718, 245.0794, 125.0251	2	0.11	0.31
Rhamnetin	$C_{16}H_{12}O_7$	316.0583	13.18	2	$[M+H]^+$	LC-QTOF-MS	ESI +	245.0434, 217.0489, 153.0191	2	0.01	0.01
Diosmetin	$C_{16}H_{12}O_6$	300.0634	13.31	1	$[M-H]^-$	LC-QTOF-MS	ESI -	299.0559, 284.0327, 266.9895	2	-	-
Epicatechin	$C_{15}H_{14}O_6$	290.0790	9.68	2	$[M-H]^-$	LC-QTOF-MS	ESI -	289.0673, 245.0828, 221.0824, 203.0727, 165.0173, 151.0397, 123.0444	2	0.01	0.02
Hyperoside	$C_{21}H_{20}O_{12}$	464.0955	12.35	1	$[M-H]^-$	LC-QTOF-MS	ESI -	464.0924, 46.0886	2	0.31	0.50
Isovitexin	$C_{21}H_{20}O_{10}$	432.1057	11.94	1	$[M-H]^-$	LC-QTOF-MS	ESI -	431.0987, 341.0668, 311.0564	2	0.04	0.08
Kaempferol-O-glucoside	$C_{21}H_{20}O_{11}$	448.1006	12.44	2	$[M-H]^-$	LC-QTOF-MS	ESI -	447.0928	2	0.01	0.01
Luteolin	$C_{15}H_{10}O_6$	286.0477	17.15	2	$[M-H]^-$	LC-QTOF-MS	ESI -	285.0399	3	0.01	0.02

Manghaslin	$C_{33}H_{40}O_{20}$	756.2113	10.50	1	$[M-H]^-$	LC-QTOF-MS	ESI -	755.2035	2	0.30	0.67
Naringenin O-glucoside	$C_{21}H_{22}O_{10}$	434.1213	13.96	1	$[M-H]^-$	LC-QTOF-MS	ESI -	433.0433	2	0.02	0.02
Pinocembrin	$C_{15}H_{12}O_4$	256.0736	18.57	1	$[M-H]^-$	LC-QTOF-MS	ESI -	255.0660, 213.0556, 211.0765, 151.0040	2	0.64	0.08
Catechin	$C_{15}H_{14}O_6$	290.0790	7.23	3	$[M-H]^-$ /$[M+H]^+$	LC-QTOF-MS	ESI -/ESI +	289.0673, 245.0828, 221.0824, 203.0727, 165.0173, 151.0397, 123.0444	2	0.01	0.02
Glycerolipids											
Monopalmitin	$C_{19}H_{38}O_4$	330.2770	23.42	-	-	GC-QTOF-MS	-	371.2983, 239.2372, 147.0656, 73.0469	2	0.67	0.68
Monostearin	$C_{21}H_{42}O_4$	358.3083	24.83	-	-	GC-QTOF-MS	-	399.3308, 361.1698, 207.0323, 147.0656, 73.0476	2	0.11	0.29
Glycols											
Diethylene Glycol	$C_4H_{10}O_3$	106.0630	9.56	-	-	GC-QTOF-MS	-	147.0656, 117.0730, 73.0464	2	0.12	0.12
Hydroxy acids and derivatives											
Glycolic Acid	$C_2H_4O_3$	76.0160	7.09	-	-	GC-QTOF-MS	-	217.1075, 147.0656, 73.0464	2	0.01	0.01
Ketoglutaric Acid	$C_5H_6O_5$	146.0215	13.92	-	-	GC-QTOF-MS	-	-	2	0.15	0.08
Lactic Acid	$C_3H_6O_3$	90.0317	6.88	-	-	GC-QTOF-MS	-	207.0323, 181.0903, 147.0656, 73.0464	2	0.16	3.80
Malic Acid	$C_4H_6O_5$	134.0215	12.89	-	-	GC-QTOF-MS	-	233.1026, 189.1129, 147.0656, 73.0476	2	3.54	3.51
Pyruvic Acid	$C_3H_4O_3$	88.0160	6.71	-	-	GC-QTOF-MS	-	-	2	0.01	-
Gulonic Acid	$C_6H_{12}O_7$	196.0583	1.16	2	$[M-H]^-$	LC-QTOF-MS	ESI -	83.0138	2	0.87	0.73
Hydroxybutyric Acid	$C_4H_8O_3$	104.0473	8.36	-	-	GC-QTOF-MS	-	233.1031, 191.0917, 147.0656, 117.0726, 66.0211	2	0.02	-
Indoles											
Formyl Indole	C_9H_7NO	145.0528	6.92	4	$[M+H]^+$	LC-QTOF-MS	ESI +	146.0606	2	0.14	0.16
Methyl esters											
Methyl benzoate	$C_8H_8O_2$	136.0524	9.00	4	$[M-H]^-$	LC-QTOF-MS	ESI -	135.0297	2	0.50	0.01
Organic acids and derivatives											
Aspartic Acid	$C_4H_7NO_4$	133.0375	13.28	-	-	GC-QTOF-MS	-	160.0792, 130.0319, 73.0464	2	0.03	0.02
Oxepanes											
Anhydro-Glucose	$C_6H_{10}O_5$	162.0528	15.19	-	-	GC-QTOF-MS	-	-	2	0.03	-
Phenolic acids and derivatives											
Caffeic Acid	$C_9H_8O_4$	180.0423	8.07	3	$[M+H-H_2O]^+$	LC-QTOF-MS	ESI +	179.0360, 135.0458	2	0.02	0.11
Dimethoxyphenol	$C_8H_{10}O_3$	154.0630	5.09	3	$[M+H-H_2O]^+$	LC-QTOF-MS	ESI +	137.0708	2	0.05	0.07
Hydroxyacetophenone	$C_8H_8O_2$	136.0524	8.29	4	$[M+H-H_2O]^+$	LC-QTOF-MS	ESI +	119.0602	2	0.02	0.02

Dihydrocaffeic acid O-glucuronide	$C_{15}H_{18}O_{10}$	358.0900	1.15	3	$[M+Na]^+$	LC-QTOF-MS	ESI +	-	2	0.16	0.13
Salicylic Acid	$C_7H_6O_3$	138.0317	4.39	3	$[M-H]^-$	LC-QTOF-MS	ESI -	93.0349	2	0.03	0.09
Pyridines and derivatives											
Nicotinic Acid	$C_6H_5NO_2$	123.0320	10.24	-	-	GC-QTOF-MS	-	180.0475, 136.0576, 106.0290, 75.0260	2	0.00	-

* For the metabolites identified by GC-QTOF-MS, the main fragment ions reported correspond to the spectrum of the derivatized metabolite, which is the form that the analyte adopts during the analysis and the one that is effectively detected by the instrument. * The relative area percentages (% RA: relative area) were calculated considering the features that presented an area value greater than $1x10^5$.

Table S2. Metabolomic profile *Euterpe oleracea* and *Euterpe precatoria* (pulp samples).

**Compound name**	**Molecular formula**	**Molecular weight (g/mol)**	**RT (min)**	**Mass error (ppm)**	**Adduct**	**Analytical platform**	**DET**	**Main fragment ions (MS/MS)**	**Identification level**	***E. oleracea ripe* (%RA)**	***E. oleracea intermediate* (%RA)**	***E. oleraceae unripe* (%RA)**	***E. precatoria ripe* (%RA)**	***E. precatoria unripe* (%RA)**
Alcohols and polyols														
Butanetriol	$C_4H_{10}O_3$	106.0630	10.31	-	-	GC-QTOF-MS	-	147.0655, 117.0728, 73.0468	2	-	-	-	0.06	0.06
Anhydroglucitol	$C_6H_{12}O_5$	164.0685	16.30	-	-	GC-QTOF-MS	-	117.0730, 73.0464	2	-	-	-	-	0.04
Hydroxymethylcatechol	$C_7H_8O_3$	140.0473	2.74	4	$[M+H-H_2O]^+$	LC-QTOF-MS	ESI +	123.0551	2	0.08	0.09	0.20	0.05	0.42
Myo-Inositol	$C_6H_{12}O_6$	180.0634	19.54	-	-	GC-QTOF-MS	-	305.1429, 217.1075, 191.0922, 147.0656, 73.0464	2	0.56	1.19	2.74	0.13	0.14
Quinic acid	$C_7H_{12}O_6$	192.0634	8.10	3	$[M-H]^-$	LC-QTOF-MS/GC-QTOF-MS	ESI -	191.0554, 127.0390, 85.0290	2	1.13	2.18	1.49	0.12	1.98
Shikimic acid	$C_7H_{10}O_5$	174.0528	1.24	4	$[M-H]^-$	LC-QTOF-MS/GC-QTOF-MS	ESI -	93.0349, 73.0296	2	0.17	0.33	0.75	0.38	0.84
Triethylene Glycol	$C_6H_{14}O_4$	150.0892	13.01	-	-	GC-QTOF-MS	-	117.0726, 116.0648, 101.0415, 75.0259, 73.0466	2	0.11	0.22	0.14	0.13	0.12
Arabitol	$C_5H_{12}O_5$	152.0685	15.51	-	-	GC-QTOF-MS	-	204.0996, 147.0656, 73.0469	2	-	0.10	0.06	-	0.08
Galactinol	$C_{12}H_{22}O_{11}$	342.1162	26.56	-	-	GC-QTOF-MS	-	281.0522, 207.0339, 147.0667, 73.0475	2	-	-	0.39	0.16	-
Mannitol	$C_6H_{14}O_6$	172.0790	17.67	-	-	GC-QTOF-MS	-	-	2	5.92	3.62	16.60	3.74	2.76
Sorbitol	$C_6H_{14}O_6$	182.0790	18.09	-	-	GC-QTOF-MS	-	319.1573, 205.1086, 147.0656, 73.0464	2	-	-	-	-	0.12
Xylitol	$C_5H_{12}O_5$	152.0685	15.26	-	-	GC-QTOF-MS	-	217.1075, 147.0656, 103.0572, 73.0464	2	-	-	0.04	0.10	0.07
Aldehydes and derivatives														
Dihydroxybenzaldehyde	$C_7H_6O_3$	138.0317	7.22	3	$[M-H]^-$ /$[M+H]^+$	LC-QTOF-MS	ESI -/ESI +	91.0189, 65.0033, 41.0043	2	0.10	0.13	0.11	0.31	1.30
Sinapoyl aldehyde	$C_{11}H_{12}O_4$	208.0736	13.59	4	$[M+H]^+$	LC-QTOF-MS	ESI +	207.0640, 192.0410, 177.0176	2	0.30	0.27	0.23	-	-
Amines														
Amino-phenylethanol	$C_8H_{11}NO$	137.0841	4.60		$[M+H-H_2O]^+$	LC-QTOF-MS	ESI +	120.0919	2	1.82	4.07	3.89	0.61	1.95
Octopamine	$C_8H_{11}NO_2$	153.079	2.74	3	$[M+H-H_2O]^+$	LC-QTOF-MS	ESI +	136.0868	2	0.15	0.19	0.36	0.09	0.78

Ethanolamine	C_2H_7NO	61.0528	9.93	-	-	GC-QTOF-MS	-	175.1134, 174.1125, 147.0652, 86.0418, 73.0466	2	-	0.23	0.29	0.11	-
Amino acids, peptides, and analogues														
Acetyl-Leucine	$C_8H_{15}NO_3$	173.1052	5.24	3	$[M+HCOOH-H]^-$	LC-QTOF-MS	ESI -	-	3	0.02	0.08	0.05	0.05	0.11
Alanine	$C_3H_7NO_2$	89.0477	7.51	-	-	GC-QTOF-MS	-	147.0651, 116.0887, 75.0258, 73.0465, 59.0309	2	0.46	0.75	0.35	0.97	0.47
Aminobutyric Acid	$C_4H_9NO_2$	103.0633	13.38	-	-	GC-QTOF-MS	-	174.1129, 147.0656, 73.0469	2	0.14	0.51	0.66	0.30	0.65
Dipeptide (Asparaginyl-Tryptophan)	$C_{15}H_{18}N_4O_4$	318.1328	5.05	0	$[M+Na]^+$	LC-QTOF-MS	ESI +	-	3	0.09	0.14	0.17	0.01	0.03
Betaine	$C_5H_{11}NO_2$	117.0790	1.18	7	$[M+H]^+$	LC-QTOF-MS	ESI +	118.0864, 59.0733, 58.0656	2	0.17	0.40	0.22	0.06	0.07
Tyrosine	$C_9H_{11}NO_3$	181.0739	2.74	3	$[M+H]^+$	LC-QTOF-MS	ESI +	136.0755, 123.0450, 119.0498, 95.0509, 91.0542	2	0.11	0.14	0.22	0.08	0.59
Glutamic Acid	$C_5H_9NO_4$	147.0532	14.45	-	-	GC-QTOF-MS	-	246.1337, 156.0836, 147.0653, 128.0888, 73.0466	2	-	-	-	0.06	-
Isoleucine	$C_6H_{13}NO_2$	131.0946	10.00	-	-	GC-QTOF-MS	-	218.1046, 158.1372, 142.1048, 73.0471	2	0.06	0.16	0.14	0.07	0.07
Isoleucylproline	$C_{11}H_{20}N_2O_3$	228.1474	3.45	5	$[M+H]^+$	LC-QTOF-MS	ESI +	-	3	0.19	0.40	0.20	0.07	0.12
Leucine	$C_6H_{13}NO_2$	131.0946	10.30	-	-	GC-QTOF-MS	-	147.0668, 73.0471	2	-	0.13	0.13	-	-
Norvaline	$C_5H_{11}NO_2$	117.0790	9.20	-	-	GC-QTOF-MS	-	174.1203, 95.0366, 73.0469	2	0.07	0.19	0.17	0.03	0.10
Phenylalanine	$C_9H_{11}NO_2$	165.0790	4.60	4	$[M+H]^+$	LC-QTOF-MS/GC-QTOF-MS	ESI +	166.0865, 120.0807, 103.0545, 79.0554	2	0.29	0.56	0.43	0.11	0.46
Dipeptide (Phenylalanyl-Glycine)	$C_{11}H_{14}N_2O_3$	222.1004	6.91	3	$[M+H-H_2O]^+$	LC-QTOF-MS	ESI +	-	3	0.18	0.42	0.35	0.06	0.21
Proline	$C_5H_9NO_2$	115.0633	10.35	-	-	GC-QTOF-MS	-	103.0207, 75.0258, 73.0466	2	0.03	0.06	0.05	0.05	-
Serine	$C_3H_7NO_3$	105.0426	9.77	-	-	GC-QTOF-MS	-	147.0656, 132.0837, 116.0532, 73.0464	2	0.06	0.10	0.05	0.14	0.14
Threonine	$C_4H_9NO_3$	119.0582	11.61	-	-	GC-QTOF-MS	-	142.1037, 130.0681, 73.0464	2	0.05	0.06	0.03	0.06	0.02
Benzoic acids and derivatives														
Hydroxybenzoic Acid	$C_7H_6O_3$	138.0317	14.49	-	-	GC-QTOF-MS	-	267.0870, 223.0972, 193.0678	2	0.01	0.01	0.01	0.02	0.02
Benzoic Acid	$C_7H_6O_2$	122.0368	9.59	-	-	GC-QTOF-MS	-	179.0528, 77.0383, 73.0464	2	0.01	0.02	0.01	0.01	0.01
Gallic Acid	$C_7H_6O_5$	170.0215	18.10	-	-	GC-QTOF-MS	-	281.0660, 73.0468	2	0.09	0.10	0.07	0.11	0.15
Protocatechoic Acid	$C_7H_6O_4$	154.0266	16.66	-	-	GC-QTOF-MS	-	-	2	-	-	-	0.01	-
Vanillic Acid	$C_8H_8O_4$	168.0423	5.45	2	$[M-H]^-$	LC-QTOF-MS	ESI -	167.0368, 152.0118, 108.0219	2	1.50	1.20	0.36	0.70	1.12
Carbohydrates and carbohydrate conjugates														
Cellobiose	$C_{12}H_{22}O_{11}$	342.1162	24.29	-	-	GC-QTOF-MS	-	-	2	-	-	-	0.27	-
Fructose	$C_6H_{12}O_6$	180.0634	17.46	-	-	GC-QTOF-MS	-	307.1579, 217.1076, 103.0575, 73.0469	2	0.84	0.13	10.22	2.21	0.97
Galactose	$C_6H_{12}O_6$	180.0634	17.61	-	-	GC-QTOF-MS	-	319.1573, 205.1066, 147.0656, 73.0464	2	0.33	0.49	2.10	0.33	0.55

Galacturonic Acid	$C_6H_{10}O_7$	194.0427	18.15	-	-	GC-QTOF-MS	-	333.1374, 160.0788, 73.0469	2	0.21	0.38	-	0.55	0.88
Gluconolactone	$C_6H_{10}O_6$	178.0477	17.47	-	-	GC-QTOF-MS	-	319.1576, 220.0946, 147.0657, 73.0469	2	-	0.45	-	1.07	1.67
Glucose	$C_6H_{12}O_6$	180.0634	17.54	-	-	GC-QTOF-MS	-	-	2	0.07	-	0.20	-	-
Glucuronic Acid	$C_6H_{10}O_7$	194.0427	18.15	-	-	GC-QTOF-MS	-	364.1802, 307.1586, 217.1095, 147.0656, 103.0572, 73.0476	2	-	-	0.14	-	-
Glycerol	$C_3H_8O_3$	92.0476	10.04	-	-	GC-QTOF-MS	-	205.1082, 147.0668, 73.0471	2	0.26	0.68	0.88	0.32	0.46
Isomaltose	$C_{12}H_{22}O_{11}$	342.1162	25.53	-	-	GC-QTOF-MS	-	-	2	-	-	-	0.23	-
Lactulose	$C_{12}H_{22}O_{11}$	342.1162	24.27	-	-	GC-QTOF-MS	-	361.1686, 204.0998, 73.0469	2	0.18	0.43	0.34	-	2.47
Maltose	$C_{12}H_{22}O_{11}$	342.1162	24.41	-	-	GC-QTOF-MS	-	361.1685, 204.0996, 73.0469	2	-	-	0.16	0.16	0.09
Palatinose	$C_{12}H_{22}O_{11}$	342.1162	25.54	-	-	GC-QTOF-MS	-	361.1684, 312.1209, 217.1073, 147.0657, 73.0469	2	-	-	-	-	0.37
Ribose	$C_5H_{10}O_5$	150.0528	15.03	-	-	GC-QTOF-MS	-	217.1075, 147.0656, 103.0572, 73.0464	2	0.12	0.30	0.13	0.16	0.09
Saccharic Acid (Glucaric Acid)	$C_6H_{10}O_8$	210.0376	18.34	-	-	GC-QTOF-MS	-	333.1372, 147.0656, 73.0469	2	0.04	0.08	0.07	0.15	0.17
Sorbose	$C_6H_{12}O_6$	180.0634	15.39	-	-	GC-QTOF-MS	-	-	2	-	0.06	-	0.02	-
Fucose	$C_6H_{12}O_5$	164.0685	2.85	3	$[M+H-H_2O]^+$	LC-QTOF-MS/GC-QTOF-MS	ESI +	137.9876, 113.9643	2	0.15	0.48	0.32	0.16	0.38
Carboxylic acids and derivatives														
Hydroxyphenyl-2-propionic Acid	$C_9H_{10}O_3$	166.063	7.34	3	$[M+H]^+$	LC-QTOF-MS	ESI +	167.0697, 122.0364, 95.0485	2	0.29	0.61	0.31	0.44	0.90
(1-Carboxy-ethyl)-3,5-cyclo-hexadiene-1,2-diol	$C_9H_{12}O_4$	184.0736	4.19	2	$[M+H-H_2O]^+$	LC-QTOF-MS	ESI +	167.0814	2	0.28	0.23	0.26	0.11	0.18
Citric Acid	$C_6H_8O_7$	192.0270	16.75	-	-	GC-QTOF-MS	-	465.1608, 375.1107, 347.1167, 273.0992, 183.0480, 147.0673, 73.0476	2	0.35	0.40	0.05	0.52	0.40
Fumaric Acid	$C_4H_4O_4$	116.0110	10.95	-	-	GC-QTOF-MS	-	-	2	-	-	0.01	-	-
Glycine	$C_2H_5NO_2$	75.0320	10.49	-	-	GC-QTOF-MS	-	248.1312, 174.1127, 147.0655, 73.0469	2	-	-	-	-	0.20
Isonicotinic Acid	$C_6H_5NO_2$	123.0320	1.24	5	$[M+H]^+$	LC-QTOF-MS	ESI +	124.0394, 96.0433, 80.0495	2	1.05	1.28	0.63	0.60	0.45
Oxalic Acid	$C_2H_2O_4$	89.9953	7.94	-	-	GC-QTOF-MS	-	147.0656, 73.0469	2	-	0.05	0.02	-	0.03
Phenylacetic Acid	$C_8H_8O_2$	136.0524	2.74	3	$[M+H-H_2O]^+$	LC-QTOF-MS	ESI +	107.0493, 95.0486, 79.0541, 77.0382	2	0.05	0.06	0.12	0.03	0.26
Succinic Acid	$C_4H_6O_4$	118.0266	10.52	-	-	GC-QTOF-MS	-	147.0656, 73.0464	2	0.21	0.35	0.33	0.12	0.13
Fatty acids and derivatives														
HODE	$C_{18}H_{32}O_3$	296.2351	18.30	2	$[M+HCOOH-H]^-$	LC-QTOF-MS	ESI -	-	3	0.53	1.06	0.87	0.15	0.54
Ethylhexanoic Acid	$C_8H_{16}O_2$	144.1150	7.41	-	-	GC-QTOF-MS	-	201.1312, 147.0656, 73.0464	2	0.13	0.21	0.21	0.17	0.18
Caprylic Acid	$C_8H_{16}O_2$	144.1150	9.77	-	-	GC-QTOF-MS	-	201.1302, 132.0837, 116.0526, 73.0468	2	0.16	0.07	0.05	-	0.22
Elaidic Acid	$C_{18}H_{34}O_2$	282.2559	20.35	-	-	GC-QTOF-MS	-	339.2744, 145.0695, 117.0381, 95.0856, 75.0267	2	0.12	-	-	-	-

Heptadecanoic Acid	$C_{17}H_{34}O_2$	270.2559	19.69	-	-	GC-QTOF-MS	-	-	2	0.02	-	0.01	-	-
Heptanoic Acid	$C_7H_{14}O_2$	130.0994	8.42	-	-	GC-QTOF-MS	-	147.0656, 117.0371, 75.0264	2	0.03	0.08	0.06	0.04	0.02
Hexanoic Acid (Caproic Acid)	$C_6H_{12}O_2$	116.0837	7.00	-	-	GC-QTOF-MS	-	173.0990, 117.0367, 75.0261	2	0.05	0.08	0.05	0.05	0.05
Hydroxyisovalerate	$C_5H_{10}O_3$	118.0630	9.08	-	-	GC-QTOF-MS	-	171.0668, 147.0668, 131.0892, 117.0366, 95.0370, 75.0260	2	0.01	0.03	0.01	0.01	-
Lactobionic Acid	$C_{12}H_{22}O_{12}$	358.1111	24.42	-	-	GC-QTOF-MS	-	361.1682, 204.0996, 147.0656, 73.0469	2	-	0.07	-	0.33	0.47
Lauric Acid	$C_{12}H_{24}O_2$	200.1776	14.70	-	-	GC-QTOF-MS	-	257.1930, 191.0916, 129.0369, 117.0367, 73.0468	2	0.04	0.07	0.05	0.04	0.03
LPI 16:0	$C_{25}H_{49}O_{12}P$	572.2962	18.87	2	$[M-H]^-$	LC-QTOF-MS	ESI -	-	3	0.06	0.07	0.13	0.09	1.92
Myristic Acid	$C_{14}H_{28}O_2$	228.2089	16.83	-	-	GC-QTOF-MS	-	285.2247, 129.0367, 117.0366, 75.0261	2	0.07	0.12	0.08	0.06	0.07
Octadecenamide (Oleamide)	$C_{18}H_{35}NO$	281.2719	18.53	4	$[M+H]^+$	LC-QTOF-MS	ESI +	-	3	0.23	0.55	0.46	0.45	0.77
Oleic Acid	$C_{18}H_{34}O_2$	282.2559	20.34	-	-	GC-QTOF-MS	-	207.0323, 147.0656, 117.0356, 75.0252	2	-	0.69	-	-	-
Palmitic Acid	$C_{16}H_{32}O_2$	256.2402	18.77	-	-	GC-QTOF-MS	-	313.2577, 132.0683, 117.0371, 73.0464	2	1.72	3.16	2.18	1.71	1.78
Stearic Acid	$C_{18}H_{36}O_2$	284.2715	20.56	-	-	GC-QTOF-MS	-	341.2877, 145.0680, 117.0368, 73.0469	2	2.39	4.71	3.05	2.59	2.59
Heptanol	$C_7H_{16}O$	116.1201	6.22	-	-	GC-QTOF-MS	-	117.0740, 75.0260, 52.0308	2	-	-	-	-	0.07
Octadecanol	$C_{18}H_{38}O$	270.2923	19.79	-	-	GC-QTOF-MS	-	327.3077, 97.1014, 75.0261, 57.0700	2	0.04	0.08	0.05	0.06	0.06
Flavonoids														
2',6'-Dihydroxy-4'-methoxydihydrochalcone	$C_{16}H_{16}O_4$	272.1049	17.93	2	$[M+H]^+$	LC-QTOF-MS	ESI +	273.0784, 153.0159, 147.1191	2	-	-	-	-	-
3,7-Dihydroxy-5,3',4'-trimethoxyflavone	$C_{18}H_{16}O_7$	344.0896	18.62	2	$[M-H]^-$	LC-QTOF-MS	ESI -	343.0816, 328.0592, 313.0352	2	0.40	1.41	1.57	-	0.01
Catechin	$C_{15}H_{14}O_6$	290.0790	8.21	2	$[M-H]^-$ /$[M+H]^+$	LC-QTOF-MS	ESI -/ESI +	289.0673, 245.0828, 221.0824, 203.0727, 165.0173, 151.0397, 123.0444	2	0.99	1.91	2.74	3.67	7.26
Cyanidin-O-galactoside	$C_{21}H_{21}O_{11}$	449.1084	8.43	2	$[M-H]^-$ /$[M+H]^+$	LC-QTOF-MS	ESI -/ESI +	450.1162, 448.1006	2	12.46	10.96	0.08	10.92	3.75
Diosmetin	$C_{16}H_{12}O_6$	300.0634	13.31	1	$[M-H]^-$	LC-QTOF-MS	ESI -	299.0559, 284.0327, 266.9895	2	0.67	1.39	1.40	-	-
Epicatechin	$C_{15}H_{14}O_6$	290.079	9.68	2	$[M-H]^-$	LC-QTOF-MS	ESI -	289.0673, 245.0828, 221.0824, 203.0727, 165.0173, 151.0397, 123.0444	2	0.35	0.43	0.38	0.29	0.42
Fisetin	$C_{15}H_{10}O_6$	286.0477	8.45	3	M+H	LC-QTOF-MS	ESI +	287.0556, 213.0546	2	23.42	11.89	0.13	16.53	6.80
Hyperoside	$C_{21}H_{20}O_{12}$	464.0955	12.35	1	$[M-H]^-$	LC-QTOF-MS	ESI -	464.0924, 46.0886	2	0.45	0.22	0.06	0.11	0.07
Isokaempferide	$C_{16}H_{12}O_6$	300.0634	13.08	2	$[M+H]^+$	LC-QTOF-MS	ESI +	301.0703, 286.0461	2	0.05	0.13	0.14	0.02	0.06
Isovitexin	$C_{21}H_{20}O_{10}$	432.1057	11.94	1	$[M-H]^-$	LC-QTOF-MS	ESI -	431.0987, 341.0668, 311.0564	2	4.38	6.23	6.86	1.21	1.82
Kaempferol-*O*-rutinoside	$C_{27}H_{30}O_{15}$	594.1585	8.66	0	$[M-H]^-$	LC-QTOF-MS	ESI -	593.1519, 284.0315	2	19.30	8.20	-	22.75	7.03
Kaempferol-*O*-glucoside	$C_{21}H_{20}O_{11}$	448.1006	12.44	2	$[M-H]^-$	LC-QTOF-MS	ESI -	-	3	3.00	3.30	4.35	1.44	2.04

Luteolin	$C_{15}H_{10}O_6$	286.0477	17.15	2	$[M-H]^-$	LC-QTOF-MS	ESI -	-	3	0.61	0.67	2.01	0.24	0.70
Manghaslin	$C_{33}H_{40}O_{20}$	756.2113	10.50	1	$[M-H]^-$	LC-QTOF-MS	ESI -	755.2035	2	0.01	0.02	0.02	0.02	0.03
Naringenin-*O*-glucoside	$C_{21}H_{22}O_{10}$	434.1213	13.96	1	$[M-H]^-$	LC-QTOF-MS	ESI -	433.1148, 271.0597, 151.0033, 107.0494	2	0.25	0.26	0.29	0.71	1.34
Pinocembrin	$C_{15}H_{12}O_4$	256.0736	18.57	1	$[M-H]^-$	LC-QTOF-MS	ESI -	255.0660, 213.0556, 211.0765, 151.0040	2	0.03	0.03	0.05	0.03	0.02
Procyanidin B2	$C_{30}H_{26}O_{12}$	578.1424	7.35	2	$[M-H]^-$ /$[M+H]^+$	LC-QTOF-MS	ESI -/ESI +	541.1018, 425.0862, 407.0776, 289.0718, 245.0794, 125.0251	2	0.21	0.29	1.10	0.26	1.26
Methylquercetin	$C_{16}H_{12}O_7$	316.0583	13.18	2	$[M+H]^+$	LC-QTOF-MS	ESI +	317.0655, 302.0447	2	0.08	0.13	0.24	0.03	0.03
Rutin	$C_{27}H_{30}O_{16}$	610.1534	9.28	1	$[M-H]^-$	LC-QTOF-MS	ESI -	609.1467, 301.0364	2	1.96	0.73	0.33	1.32	0.20
Santin	$C_{18}H_{16}O_7$	344.0896	17.72	1	$[M-H]^-$	LC-QTOF-MS	ESI -	343.0816, 328.0592, 313.0352	2	0.69	1.56	0.80	-	-
Taxifolin	$C_{15}H_{12}O_7$	304.0583	10.11	3	$[M-H]^-$ /$[M+H]^+$	LC-QTOF-MS	ESI -/ESI +	301.0360, 273.0410, 255.0300, 125.0240	2	0.49	0.47	0.32	0.70	0.48
Glycerolipids														
Monopalmitin	$C_{19}H_{38}O_4$	330.2770	23.42	-	-	GC-QTOF-MS	-	371.2983, 239.2372, 147.0656, 73.0469	2	0.80	1.94	0.87	1.04	1.10
Monostearin	$C_{21}H_{42}O_4$	358.3083	24.84	-	-	GC-QTOF-MS	-	399.3308, 361.1698, 207.0323, 147.0656, 73.0476	2	0.37	0.73	0.50	0.41	0.29
Glycols														
Diethylene Glycol	$C_4H_{10}O_3$	106.0630	9.56	-	-	GC-QTOF-MS	-	147.0656, 117.0730, 73.0464	2	0.17	0.32	0.19	0.18	0.18
Hydroxy acids and derivatives														
Glycolic Acid	$C_2H_4O_3$	76.0160	7.09	-	-	GC-QTOF-MS	-	217.1075, 147.0656, 73.0464	2	0.02	0.03	0.03	0.02	-
Gulonic Acid	$C_6H_{12}O_7$	196.0583	1.16	2	$[M-H]^-$	LC-QTOF-MS	ESI -	83.0138	2	0.29	0.71	0.37	0.20	0.30
Lactic Acid	$C_3H_6O_3$	90.0317	6.87	-	-	GC-QTOF-MS	-	207.0323, 181.0903, 147.0656, 73.0464	2	0.16	0.77	0.36	0.16	0.29
Malic Acid	$C_4H_6O_5$	134.0215	12.89	-	-	GC-QTOF-MS	-	233.1026, 189.1129, 147.0656, 73.0476	2	0.20	1.06	0.90	0.09	0.05
Pyruvic Acid	$C_3H_4O_3$	88.0160	7.28	-	-	GC-QTOF-MS	-	-	2	-	-	-	-	0.03
Hydroxybutyric Acid	$C_4H_8O_3$	104.0473	8.35	-	-	GC-QTOF-MS	-	233.1031, 191.0917, 147.0656, 117.0726, 66.0211	2	-	-	-	-	0.01
Indoles														
Formyl Indole	C_9H_7NO	145.0528	6.92	4	$[M+H]^+$	LC-QTOF-MS	ESI +	146.0606	3	0.22	0.46	0.40	0.09	0.21
Lactones														
Dehydroascorbic Acid	$C_6H_6O_6$	174.0164	17.08	-	-	GC-QTOF-MS	-	-	2	0.06	-	0.09	-	-
Methyl esters														
Methyl benzoate	$C_8H_8O_2$	136.0524	9.00	4	$[M-H]^-$	LC-QTOF-MS	ESI -	135.0446	2	-	0.04	0.08	0.02	0.04
Oxepanes														
Levoglucosan	$C_6H_{10}O_5$	162.0528	15.52	-	-	GC-QTOF-MS	-	-	2	0.03	-	-	0.06	-

Phenolic acids and derivatives														
Dimethoxyphenol	$C_8H_{10}O_3$	154.0630	5.09	3	$[M+H-H_2O]^+$	LC-QTOF-MS	ESI +	137.0708	2	0.26	0.71	0.47	0.18	0.38
Caffeic Acid	$C_9H_8O_4$	180.0423	8.07	3	$[M-H]^-$ /$[M+H-H_2O]^+$	LC-QTOF-MS	ESI -/ESI +	179.0360, 135.0458	2	0.25	0.35	0.51	0.06	0.83
Chlorogenic Acid	$C_{16}H_{18}O_9$	354.0951	9.17	1	$[M+HCOOH-H]^-$	LC-QTOF-MS	ESI -	-	3	0.68	1.67	1.12	-	-
Coumaric Acid	$C_9H_8O_3$	164.0473	8.30	3	$[M-H]^-$	LC-QTOF-MS	ESI -	163.0409, 119.0509	2	0.65	1.65	1.06	0.44	0.54
Dihydrocaffeic Acid *O*-glucuronide	$C_{15}H_{18}O_{10}$	358.090	1.15	3	$[M+Na]^+$	LC-QTOF-MS	ESI +	-	2	0.23	0.36	0.35	0.13	0.21
Hydroxyacetophenone	$C_8H_8O_2$	136.0524	8.29	4	$[M+H-H_2O]^+$	LC-QTOF-MS	ESI +	119.0602	2	0.10	0.21	0.21	0.22	0.52
Salicylic Acid	$C_7H_6O_3$	138.0317	4.39	3	$[M-H]^-$	LC-QTOF-MS	ESI -	93.0349	2	0.34	0.23	0.42	2.03	1.85
Prenol lipids														
Squalene	$C_{30}H_{50}$	410.3913	25.15	-	-	GC-QTOF-MS	-	207.0339, 147.0668, 121.1019, 73.0471	2	0.06	0.28	0.16	-	-
Pyridines and derivatives														
Nicotinic Acid	$C_6H_5NO_2$	123.0320	10.25	-	-	GC-QTOF-MS	-	180.0475, 136.0576, 106.0290, 75.0260	2	0.01	-	-	-	-
Ureas														
Urea	CH_4N_2O	60.0324	9.41	-	-	GC-QTOF-MS	-	189.0863, 147.0656, 75.0264, 52.0302	2	0.03	0.07	0.04	-	0.03
Vitamins														
Riboflavin	$C_{17}H_{20}N_4O_6$	376.1383	3.37	1	$[M+Na]^+$	LC-QTOF-MS	ESI +	377.1476, 360.1210, 243.0883, 198.0660, 172.0877	2	0.17	0.37	0.29	0.23	0.27

* For the metabolites identified by GC-QTOF-MS, the main fragment ions reported correspond to the spectrum of the derivatized metabolite, which is the form that the analyte adopts during the analysis and the one that is effectively detected by the instrument. * The relative area percentages (% RA: relative area) were calculated considering the features that presented an area value greater than $1x10^5$.

Table S3. Metabolomic profile *Theobroma grandiflorum* (pulp and seeds).

**Compound name**	**Molecular formula**	**Molecular weight (g/mol)**	**RT (min)**	**Mass error (ppm)**	**Adduct**	**Analytical platform**	**DET**	**Main fragment ions (MS/MS)**	**Identification level**	***T. grandiflorum pulp ripe* (%RA)**	***T. grandiflorum pulp unripe* (%RA)**	***T. grandiflorum seeds ripe* (%RA)**	***T. grandiflorum seeds unripe* (%RA)**
Alcohols and polyols													
Butanediol	$C_4H_{10}O_2$	90.0681	6.46	-	-	GC-QTOF-MS	-	152.0530, 117.0730, 73.0464	2	-	0.26	0.08	-
Heptanol	$C_7H_{16}O$	116.1201	6.22	-	-	GC-QTOF-MS	-	117.0740, 75.0260, 52.0308	2	-	-	0.02	-
Lactamide	$C_3H_7NO_2$	89.0477	8.35	-	-	GC-QTOF-MS	-	191.0925, 147.0656, 73.0472	2	-	-	0.01	-
Erythritol	$C_4H_{10}O_4$	122.0579	13.19	-	-	GC-QTOF-MS	-	217.1075, 147.0656, 73.0464	2	-	0.01	0.03	0.03
Myo-Inositol	$C_6H_{12}O_6$	180.0634	19.53	-	-	GC-QTOF-MS	-	305.1429, 217.1075, 191.0922, 147.0656, 73.0464	2	7.12	3.82	1.79	2.65
Octadecanol	$C_{18}H_{38}O$	270.2923	19.77	-	-	GC-QTOF-MS	-	327.3077, 97.1014, 75.0261, 57.0700	2	-	-	-	0.01

Galactinol	$C_{12}H_{22}O_{11}$	342.1162	26.70	-	-	GC-QTOF-MS	-	281.0522, 207.0339, 147.0667, 73.0475	2	-	-	0.02	-
Sorbitol	$C_6H_{14}O_6$	182.0790	17.85	-	-	GC-QTOF-MS	-	319.1573, 205.1086, 147.0656, 73.0464	2	1.30	2.26	5.87	4.33
Xylitol	$C_5H_{12}O_5$	152.0685	15.26	-	-	GC-QTOF-MS	-	217.1075, 147.0656, 103.0572, 73.0464	2	0.02	0.05	0.02	0.15
Amines													
Ethanolamine	C_2H_7NO	61.0528	9.92	-	-	GC-QTOF-MS	-	175.1134, 174.1125, 147.0652, 86.0418, 73.0466	2	0.05	0.03	-	0.05
Amino acids, peptides, and analogues													
Alanine	$C_3H_7NO_2$	89.0477	7.50	-	-	GC-QTOF-MS	-	147.0651, 116.0887, 75.0258, 73.0465, 59.0309	2	0.08	0.24	0.33	0.39
Aminobutanoic Acid	$C_4H_9NO_2$	103.0633	13.37	-	-	GC-QTOF-MS	-	304.1581, 174.1131, 147.0656, 73.0464	2	-	0.77	0.27	0.35
Asparagine	$C_4H_8N_2O_3$	132.0535	14.12	-	-	GC-QTOF-MS	-	159.0939, 147.0656, 73.0464	2	-	0.03	0.01	0.03
Aspartic Acid	$C_4H_7NO_4$	133.0375	11.99	-	-	GC-QTOF-MS	-	160.0792, 130.0319, 73.0464	2	2.31	1.84	0.44	0.36
Cadaverine	$C_5H_{14}N_2$	102.1157	12.52	-	-	GC-QTOF-MS	-	174.1131, 73.0464	2	-	0.04	-	-
Glucosamine phosphate	$C_6H_{10}NO_9P$	271.0093	16.25	-	-	GC-QTOF-MS	-	-	2	-	0.03	0.04	0.02
Glucosaminic Acid	$C_6H_{13}NO_6$	195.0743	18.19	-	-	GC-QTOF-MS	-	361.1672, 217.1075, 147.0666, 73.0464	2	0.06	0.08	0.76	0.24
Glutamic Acid	$C_5H_9NO_4$	147.0532	14.43	-	-	GC-QTOF-MS	-	246.1337, 156.0836, 147.0653, 128.0888, 73.0466	2	0.06	0.10	0.10	0.06
Glutamine	$C_5H_{10}N_2O_3$	146.0691	16.16	-	-	GC-QTOF-MS	-	156.0835, 73.0464	2	-	-	-	0.04
Isoleucine	$C_6H_{13}NO_2$	131.0946	10.30	-	-	GC-QTOF-MS	-	218.1046, 158.1372, 142.1048, 73.0471	2	-	-	0.03	0.05
Leucine	$C_6H_{13}NO_2$	131.0946	9.99	-	-	GC-QTOF-MS	-	147.0668, 73.0471	2	-	-	-	0.13
Norleucine	$C_6H_{13}NO_2$	131.0946	9.99	-	-	GC-QTOF-MS	-	158.1359, 73.0464	2	-	-	0.03	-
Phenylalanine	$C_9H_{11}NO_2$	165.0790	14.54	-	-	GC-QTOF-MS	-	166.0865, 120.0807, 103.0545, 79.0554	2	-	0.01	0.01	0.05
Proline	$C_5H_9NO_2$	115.0633	8.54	-	-	GC-QTOF-MS	-	103.0207, 75.0258, 73.0466	2	0.13	0.05	0.05	0.13
Serine	$C_3H_7NO_3$	105.0426	9.75	-	-	GC-QTOF-MS	-	147.0656, 132.0837, 116.0532, 73.0464	2	0.12	0.10	0.09	0.10
Threonine	$C_4H_9NO_3$	119.0582	10.29	-	-	GC-QTOF-MS	-	142.1037, 130.0681, 73.0464	2	0.10	-	0.03	0.08
Valine	$C_5H_{11}NO_2$	117.0790	9.19	-	-	GC-QTOF-MS	-	144.1204, 73.0464	2	0.02	0.01	0.03	0.09
Tripeptide (Ala-Asp-Cys)	$C_{10}H17N_3O_6S$	307.0838	1.87	0	$[M-H]^-$	LC-QTOF-MS	ESI-	-	3	1.13	0.29	-	-
Benzoic acids and derivatives													
Benzoic Acid	$C_7H_6O_2$	122.0368	9.59	-	-	GC-QTOF-MS	-	179.0528, 77.0383, 73.0464	2	0.01	-	0.03	0.02
Carbohydrates and carbohydrate conjugates													
Acetyl-Mannosamine	$C_8H_{17}NO_7$	239.1005	19.98	-	-	GC-QTOF-MS	-	-	2	-	-	0.08	0.04
Amino-2-deoxyhexose	$C_6H_{13}NO_5$	179.0794	21.63	-	-	GC-QTOF-MS	-	-	2	0.02	0.02	-	-
Anhydroglucitol	$C_6H_{12}O_5$	164.0685	13.00	-	-	GC-QTOF-MS	-	117.0730, 73.0464	2	0.02	-	0.05	0.07
Deoxy-Ribose	$C_5H_{10}O_4$	134.0579	13.61	-	-	GC-QTOF-MS	-	217.1085, 189.0772, 147.0663, 117.0737, 103.0473	2	-	0.03	0.01	-

Galactose	$C_6H_{12}O_6$	180.0634	17.60	-	-	GC-QTOF-MS	-	319.1573, 205.1066, 147.0656, 73.0464	2	11.91	12.18	18.27	18.03
Gentiobiose	$C_{12}H_{22}O_{11}$	342.1162	24.99	-	-	GC-QTOF-MS	-	361.1698, 204.0997, 147.0656, 73.0464	2	-	1.54	0.41	-
Gluconic Acid	$C_6H_{12}O_7$	196.0583	17.49	-	-	GC-QTOF-MS	-	307.1586, 217.1075, 147.0656, 73.0476	2	6.90	6.40	12.57	10.86
Glucose-phosphate	$C_6H_{13}O_9P$	260.0297	21.62	-	-	GC-QTOF-MS	-	-	2	-	0.01	-	-
Glucuronic Acid	$C_6H_{10}O_7$	194.0427	17.39	-	-	GC-QTOF-MS	-	364.1802, 307.1586, 217.1095, 147.0656, 103.0572, 73.0476	2	10.92	9.91	15.85	15.88
Glyceric Acid	$C_3H_6O_4$	106.0266	10.83	-	-	GC-QTOF-MS	-	189.0768, 147.0656, 73.0464	2	0.02	0.03	0.01	0.05
Glycerol	$C_3H_8O_3$	92.0473	10.04	-	-	GC-QTOF-MS	-	205.1082, 147.0668, 73.0471	2	-	-	0.36	-
Melibiose	$C_{12}H_{22}O_{11}$	342.1162	25.71	-	-	GC-QTOF-MS	-	361.7672, 281.0501, 207.0323, 73.0464	2	-	0.05	0.03	-
Mucic Acid	$C_6H_{10}O_8$	210.0376	18.71	-	-	GC-QTOF-MS	-	333.1378, 292.1351, 217.1091, 147.0668, 73.0471	2	-	-	0.04	0.03
Ribose	$C_5H_{10}O_5$	150.0528	14.92	-	-	GC-QTOF-MS	-	217.1075, 147.0656, 103.0572, 73.0464	2	0.03	0.10	0.09	0.18
Ribulose-Phosphate	$C_5H_{11}O_8P$	230.0192	19.50	-	-	GC-QTOF-MS	-	-	2	0.41	0.14	0.09	0.17
Tartaric Acid	$C_4H_6O_6$	150.0164	14.76	-	-	GC-QTOF-MS	-	292.1353, 189.0768, 147.0656, 73.0464	2	0.02	0.02	0.01	0.01
Lactose	$C_{12}H_{22}O_{11}$	342.1162	1.24	2	$[M-H]^-$	LC-QTOF-MS	ESI -	341.1084	3	6.31	0.63	0.49	0.31
Hydroxy acids													
Glycolic Acid	$C_2H_4O_3$	76.0160	7.08	-	-	GC-QTOF-MS	-	217.1075, 147.0656, 73.0464	2	0.03	0.01	0.03	0.03
Lactic Acid	$C_3H_6O_3$	90.0317	6.87	-	-	GC-QTOF-MS	-	207.0323, 181.0903, 147.0656, 73.0464	2	0.07	0.02	0.01	0.07
Malic Acid	$C_4H_6O_5$	134.0215	12.88	-	-	GC-QTOF-MS	-	233.1026, 189.1129, 147.0656, 73.0476	2	5.02	22.47	11.09	7.14
Pyruvic Acid	$C_3H_4O_3$	88.0160	2.07	4	$[M-H]^-$	LC-QTOF-MS	ESI -	87.0082	3	1.80	1.96	0.44	1.34
Carboxylic acids and derivatives													
Aconitic Acid	$C_6H_6O_6$	174.0164	15.86	-	-	GC-QTOF-MS	-	229.1081, 147.0656, 73.0464	2	-	0.01	0.02	0.01
Butenedioic Acid	$C_4H_4O_4$	116.0110	10.94	-	-	GC-QTOF-MS	-	245.0660, 147.0656, 73.0464	2	-	0.03	0.05	0.04
Citric Acid	$C_6H_8O_7$	192.0270	16.74	-	-	GC-QTOF-MS	-	465.1608, 375.1107, 347.1167, 273.0992, 183.0480, 147.0673, 73.0476	2	35.43	24.52	13.56	12.24
Galactaric Acid	$C_6H_{10}O_8$	210.0376	19.12	-	-	GC-QTOF-MS	-	333.1379, 292.1330, 147.0656, 73.0464	2	-	0.01	-	-
Glutaric Acid	$C_5H_8O_4$	132.0423	13.91	-	-	GC-QTOF-MS	-	-	2	-	0.08	-	-
Methylmalonic Acid	$C_4H_6O_4$	118.0266	8.99	-	-	GC-QTOF-MS	-	147.0668, 75.0260	2	-	-	0.01	0.01
Oxalic Acid	$C_2H_2O_4$	89.9953	7.93	-	-	GC-QTOF-MS	-	147.0656, 73.0469	2	-	-	0.08	0.10
Oxoglutaric Acid	$C_5H_6O_5$	146.0215	13.91	-	-	GC-QTOF-MS	-	-	2	-	-	0.02	0.03
Quininic Acid	$C_{11}H_9NO_3$	203.0582	17.25	-	-	GC-QTOF-MS	-	345.1741, 255.1238, 147.0656, 73.0464	2	-	0.10	0.08	0.15
Shikimic Acid	$C_7H_{10}O_5$	174.0528	16.57	-	-	GC-QTOF-MS	-	93.0349, 73.0296	2	-	0.01	-	-
Succinic Acid	$C_4H_6O_4$	118.0266	10.51	-	-	GC-QTOF-MS	-	147.0656, 73.0464	2	0.05	0.04	0.09	0.06
Glutaconic Acid	$C_5H_6O_4$	130.0266	2.07	3	$[M-H]^-$	LC-QTOF-MS	ESI -	-	3	2.72	3.28	0.60	2.13

2-hydroxy-butanoic Acid	$C_4H_8O_3$	104.0473	2.08	7	$[M-H]^-$	LC-QTOF-MS	ESI -	-	3	1.62	1.69	0.34	1.09
Maleic Acid	$C_4H_4O_4$	116.0110	1.32	7	$[M-H]^-$	LC-QTOF-MS	ESI -	-	3	0.64	0.97	0.35	0.28
Acetyl-aspartic Acid	$C_6H_9NO_5$	175.0481	6.98	2	$[M-H]^-$	LC-QTOF-MS	ESI -	-	3	0.05	0.49	0.40	0.74
Fatty acids and conjugates													
Arachidic Acid	$C_{20}H_{40}O_2$	312.3028	22.19	-	-	GC-QTOF-MS	-	369.3208, 207.0339, 117.0381, 75.0272	2	-	-	0.02	0.01
Caprylic Acid	$C_8H_{16}O_2$	144.1150	9.78	-	-	GC-QTOF-MS	-	201.1302, 132.0837, 116.0526, 73.0468	2	-	-	0.02	0.01
Elaidic Acid	$C_{18}H_{34}O_2$	282.2559	20.33	-	-	GC-QTOF-MS	-	339.2744, 145.0695, 117.0381, 95.0856, 75.0267	2	-	-	0.16	0.12
Ethylhexanoic Acid	$C_8H_{16}O_2$	144.1150	7.39	-	-	GC-QTOF-MS	-	201.1312, 147.0656, 73.0464	2	0.08	0.04	0.05	0.04
Heptanoic Acid	$C_7H_{14}O_2$	130.0994	8.41	-	-	GC-QTOF-MS	-	147.0656, 117.0371, 75.0264	2	0.02	0.01	0.01	0.02
Hydroxyisovalerate	$C_5H_{10}O_3$	118.0630	9.08	-	-	GC-QTOF-MS	-	171.0668, 147.0668, 131.0892, 117.0366, 95.0370, 75.0260	2	-	-	0.01	-
Lauric acid	$C_{12}H_{24}O_2$	200.1776	14.69	-	-	GC-QTOF-MS	-	257.1930, 191.0916, 129.0369, 117.0367, 73.0468	2	-	-	-	0.02
Linoleic Acid	$C_{18}H_{32}O_2$	280.2402	20.30	-	-	GC-QTOF-MS	-	-	2	-	-	0.07	-
Oleic Acid	$C_{18}H_{34}O_2$	282.2559	20.33	-	-	GC-QTOF-MS	-	207.0323, 147.0656, 117.0356, 75.0252	2	0.06	-	-	-
Palmitic Acid	$C_{16}H_{32}O_2$	256.2402	18.76	-	-	GC-QTOF-MS	-	313.2577, 132.0683, 117.0371, 73.0464	2	0.58	0.26	0.49	1.05
Flavonoids													
Procyanidin B1	$C_{30}H_{26}O_{12}$	578.1424	9.03	3	$[M-H]^-$	LC-QTOF-MS	ESI -	-	3	0.85	0.52	2.04	3.75
Catechin	$C_{15}H_{14}O_6$	290.0790	9.71	1	$[M-H]^-$	LC-QTOF-MS	ESI -	289.0673, 245.0828, 221.0824, 203.0727, 165.0173, 151.0397, 123.0444	3	0.43	0.41	1.01	1.40
Epigallocatechin gallate	$C_{22}H_{18}O_{11}$	458.0849	8.76	-1	$[M-H]^-$	LC-QTOF-MS	ESI -	-	3	0.26	0.90	0.16	0.12
Procyanidin C1	$C_{45}H_{38}O_{18}$	866.2058	10.23	2	$[M-H]^-$	LC-QTOF-MS	ESI -	-	3	0.34	0.20	1.80	2.65
Quercetin glucuronide	$C_{21}H_{18}O_{13}$	478.0747	13.98	1	$[M-H]^-$	LC-QTOF-MS	ESI -	-	3	-	-	3.40	3.02
Wogonoside	$C_{22}H_{20}O_{11}$	460.1006	15.36	-3	$[M-H]^-$	LC-QTOF-MS	ESI -	-	3	-	-	1.82	1.71
Baicalin	$C_{21}H_{18}O_{11}$	446.0849	15.46	-2	$[M-H]^-$	LC-QTOF-MS	ESI -	-	3	-	-	1.27	1.41
Apigenin digalactoside	$C_{27}H_{30}O_{15}$	594.1585	10.63	2	$[M-H]^-$	LC-QTOF-MS	ESI -	-	3	-	-	0.52	0.53
Furanocoumarins													
Xanthotoxin	$C_{12}H_8O_4$	216.0423	20.54	-	-	GC-QTOF-MS	-	341.2873, 117.0371, 73.0464	2	0.90	0.35	0.78	1.53
Glycols													
Diethylene glycol	$C_4H_{10}O_3$	106.0630	9.55	-	-	GC-QTOF-MS	-	147.0656, 117.0730, 73.0464	2	-	0.04	0.03	0.05
Triethylene glycol	$C_6H_{14}O_4$	150.0892	13.00	-	-	GC-QTOF-MS	-	117.0726, 116.0648, 101.0415, 75.0259, 73.0466	2	-	0.03	-	-
Monoacylglycerols													
Monostearin	$C_{21}H_{42}O_4$	358.3083	24.82	-	-	GC-QTOF-MS	-	399.3308, 361.1698, 207.0323, 147.0656, 73.0476	2	-	-	0.16	0.24

Oleoylglycerol	$C_{21}H_{40}O_4$	356.2927	24.67	-	-	GC-QTOF-MS	-	361.1698, 207.0339, 147.0668, 73.0471	2	-	-	0.06	0.11
Stearoyl-glycerol	$C_{21}H_{42}O_4$	358.3083	24.83	-	-	GC-QTOF-MS	-	399.3280, 361.1672, 207.0323, 73.0464	2	-	0.13	0.05	-
Phosphates													
Phosphoric Acid	H_3O_4P	97.9769	10.03	-	-	GC-QTOF-MS	-	-	2	-	0.32	0.48	1.77
Triterpenoids													
Squalene	$C_{30}H_{50}$	410.3913	25.14	-	-	GC-QTOF-MS	-	207.0339, 147.0668, 121.1019, 73.0471	2	-	-	0.01	-
Ureas													
Urea	CH_4N_2O	60.0324	9.41	-	-	GC-QTOF-MS	-	189.0863, 147.0656, 75.0264, 52.0302	2	-	0.01	-	0.02

* For the metabolites identified by GC-QTOF-MS, the main fragment ions reported correspond to the spectrum of the derivatized metabolite, which is the form that the analyte adopts during the analysis and the one that is effectively detected by the instrument. * The relative area percentages (% RA-relative area) were calculated considering the features that presented an area value greater than $1x10^5$.

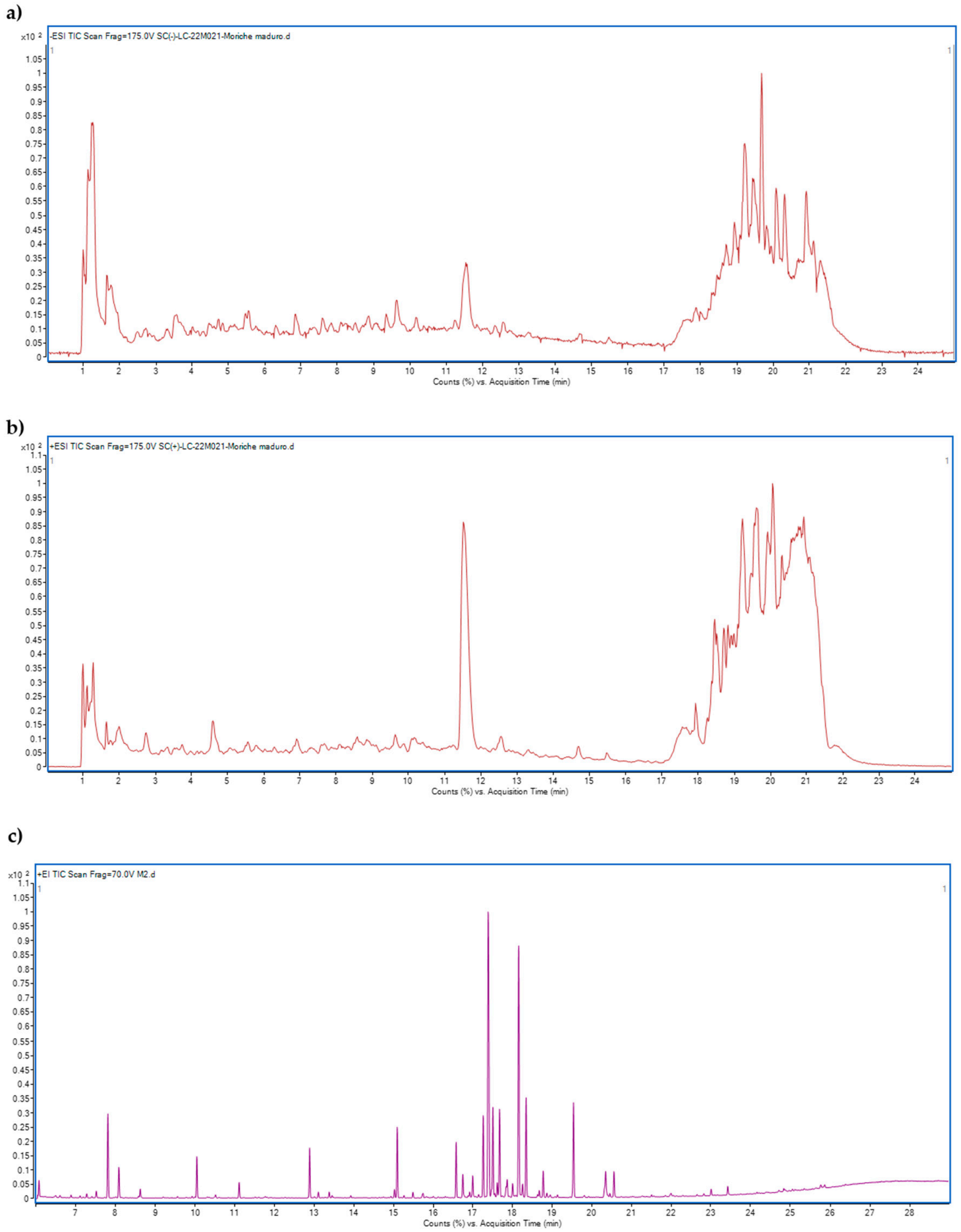

Figure S1. Chromatograms of the *Mauritia flexuosa* ripe pulp extract obtained by **a)** LC-QTOF-MS(-), **b)** LC-QTOF-MS(+), and **c)** GC-QTOF-MS.

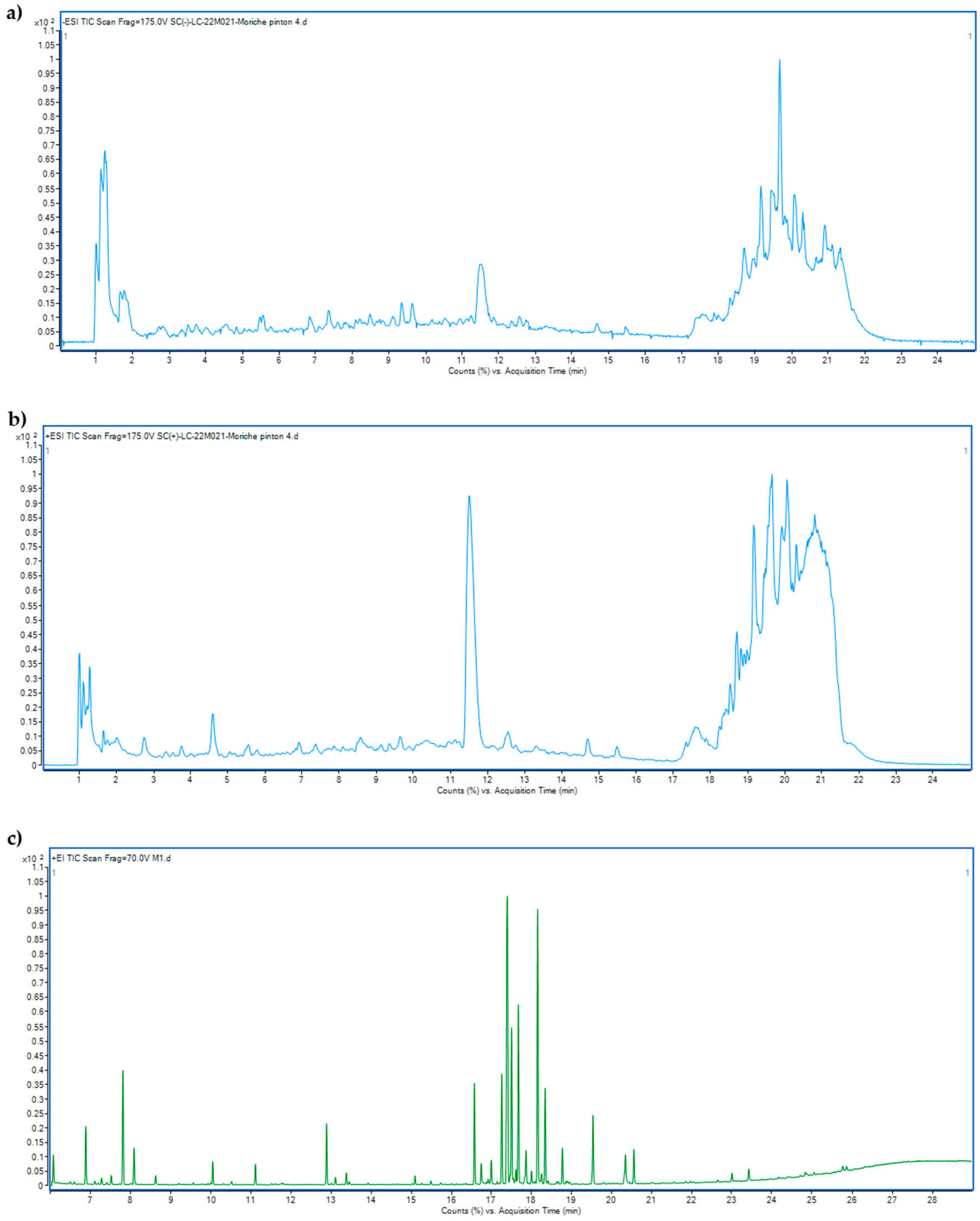

Figure S2. Chromatograms of the *Mauritia flexuosa* unripe pulp extract obtained by **a)** LC-QTOF-MS(-), **b)** LC-QTOF-MS(+), and **c)** GC-QTOF-MS.

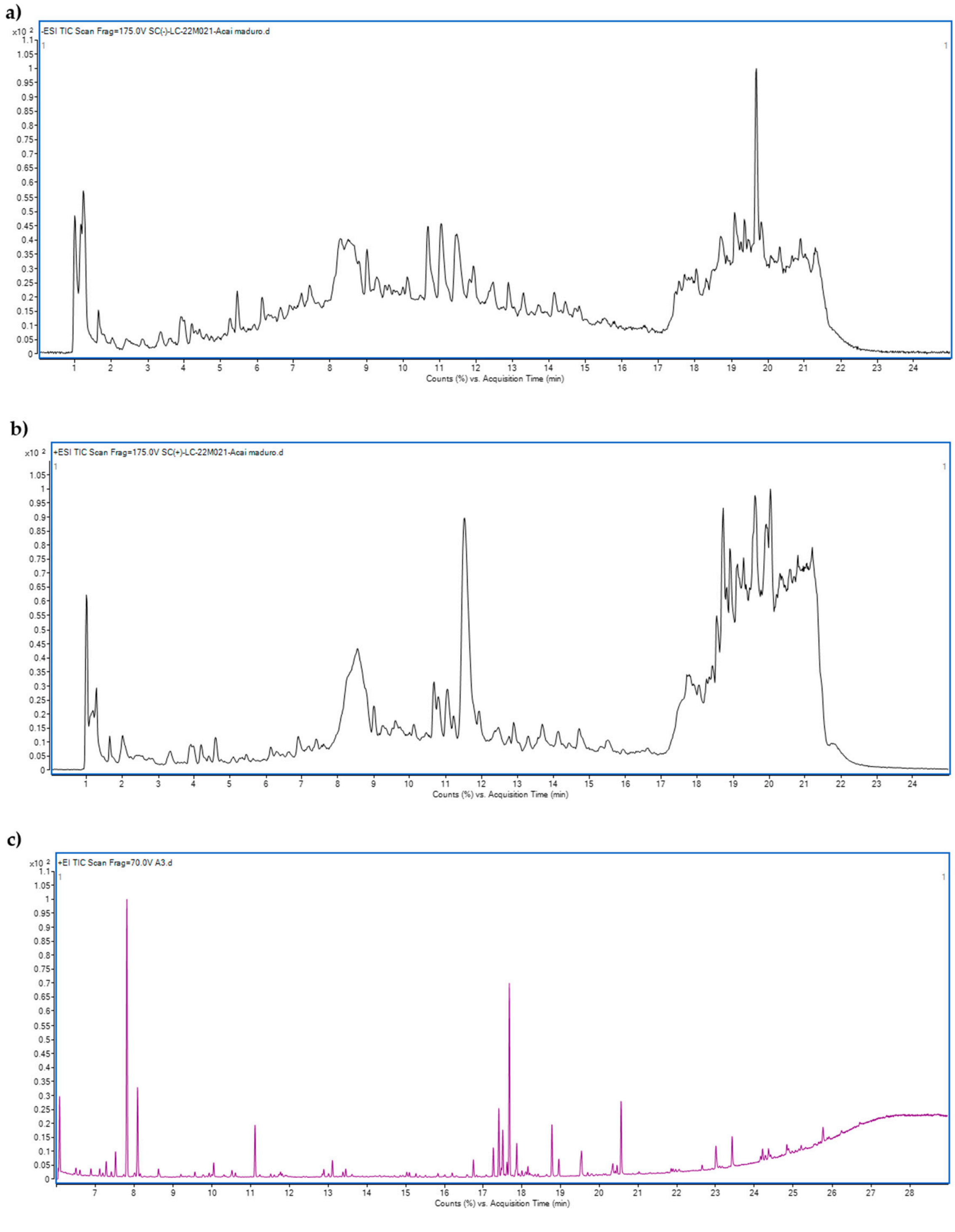

Figure S3. Chromatograms of the *Euterpe oleracea* ripe pulp extract obtained by **a)** LC-QTOF-MS(-), **b)** LC-QTOF-MS(+), and **c)** GC-QTOF-MS.

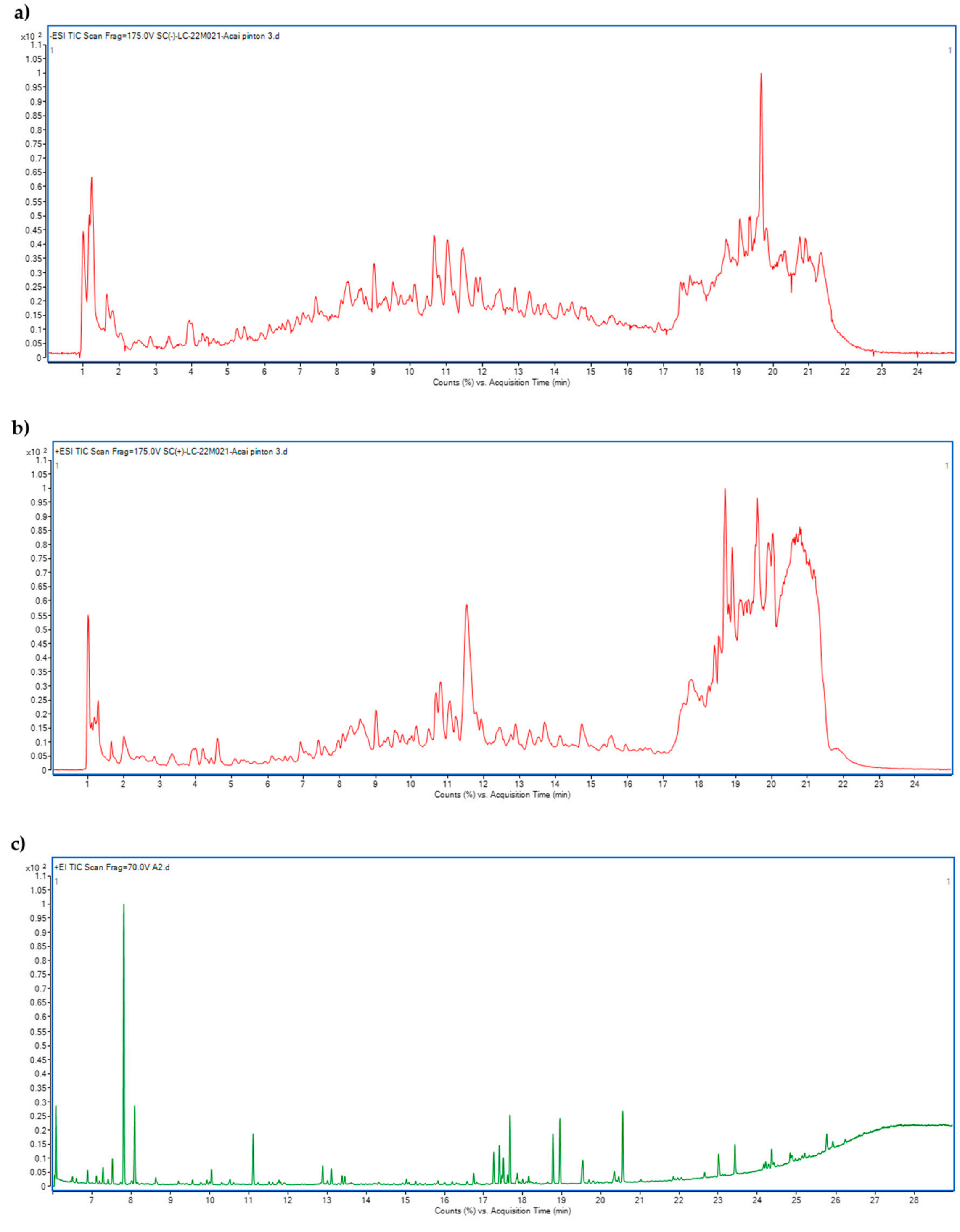

Figure S4. Chromatograms of the *Euterpe oleracea* intermediate pulp extract obtained by **a)** LC-QTOF-MS(-), **b)** LC-QTOF-MS(+), and **c)** GC-QTOF-MS.

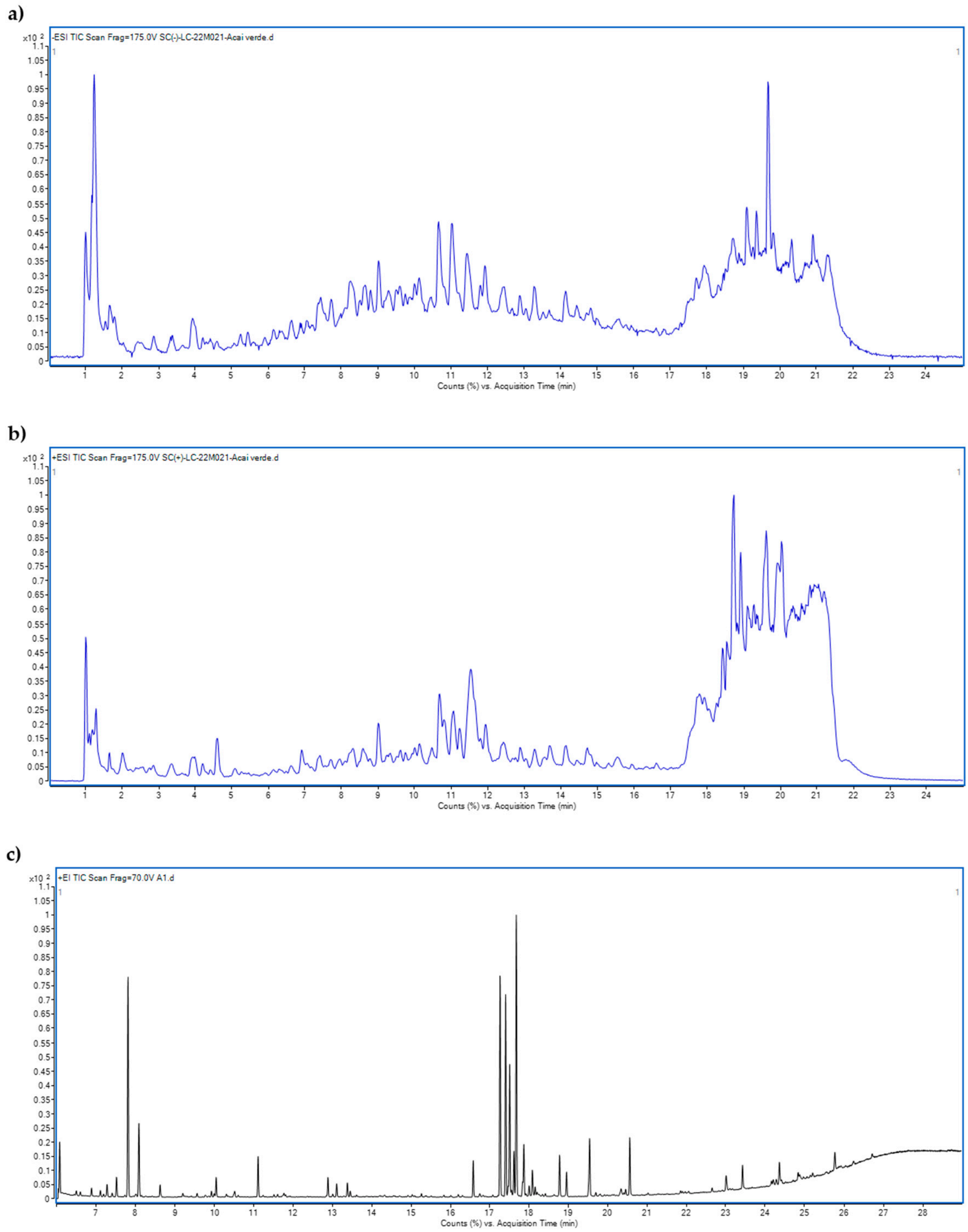

Figure S5. Chromatograms of the *Euterpe oleracea* unripe pulp extract obtained by **a)** LC-QTOF-MS(-), **b)** LC-QTOF-MS(+), and **c)** GC-QTOF-MS.

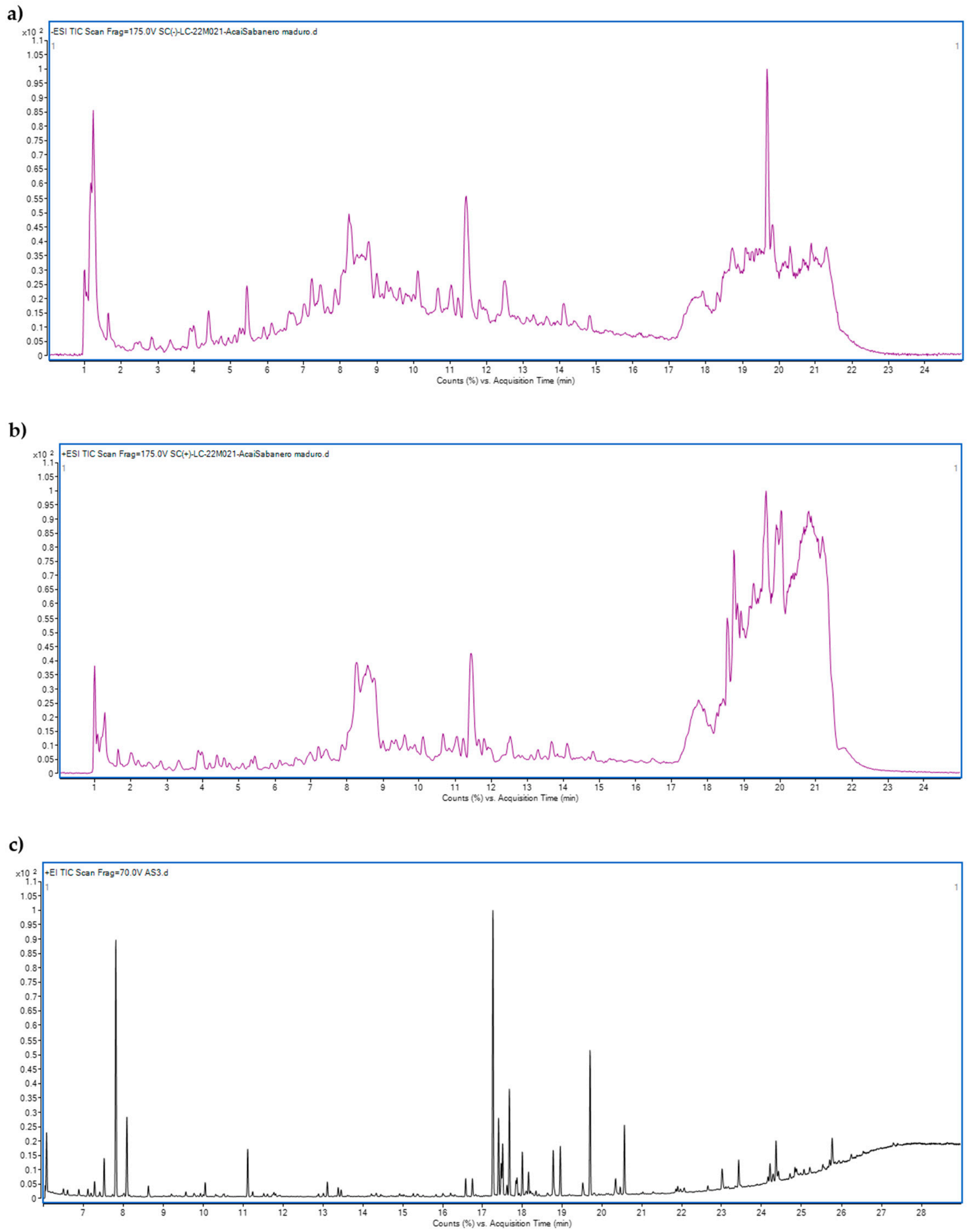

Figure S6. Chromatograms of the *Euterpe precatoria* ripe pulp extract obtained by **a)** LC-QTOF-MS(-), **b)** LC-QTOF-MS(+), and **c)** GC-QTOF-MS.

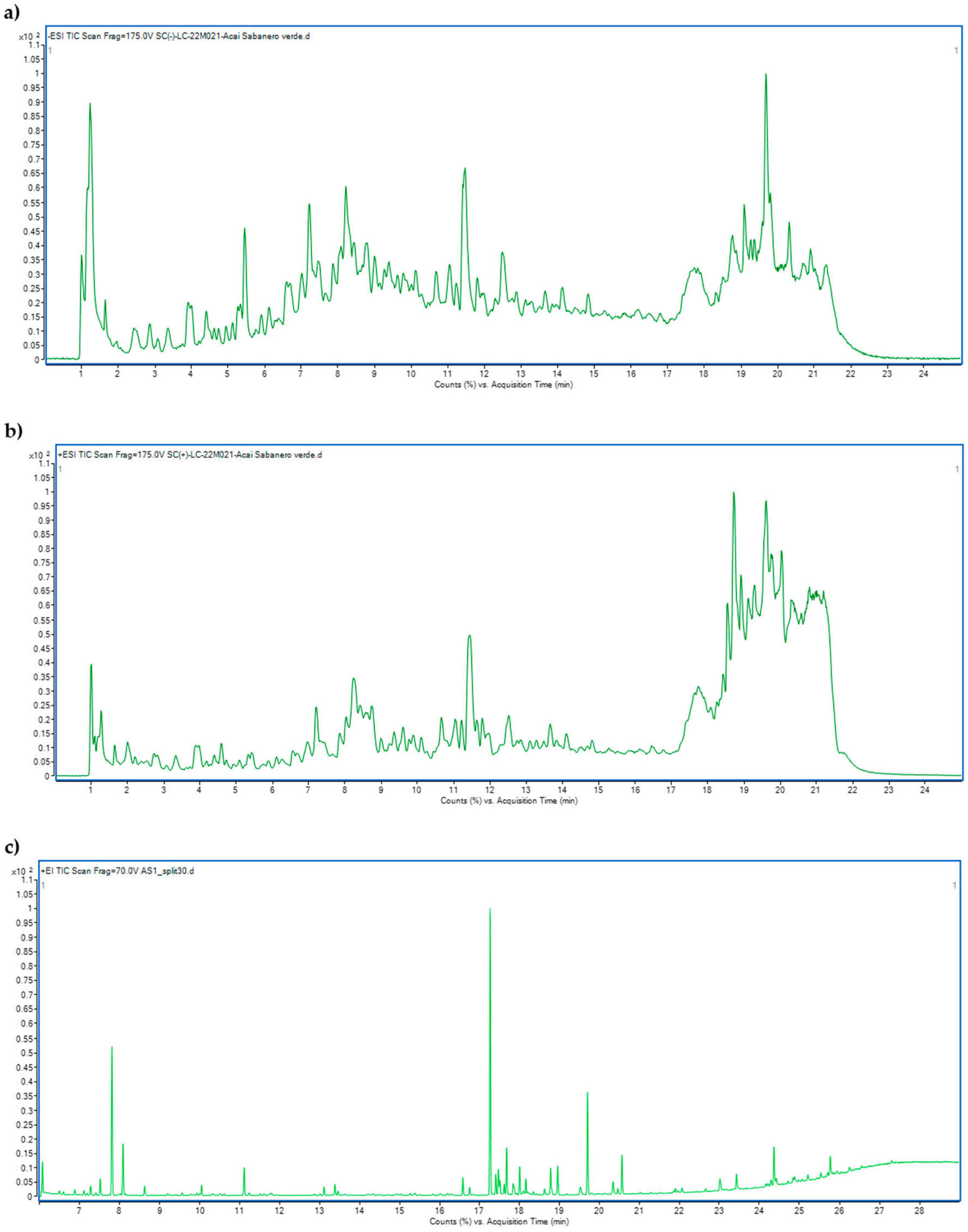

Figure S7. Chromatograms of the *Euterpe precatoria* unripe pulp extract obtained by **a)** LC-QTOF-MS(-), **b)** LC-QTOF-MS(+), and **c)** GC-QTOF-MS.

a)

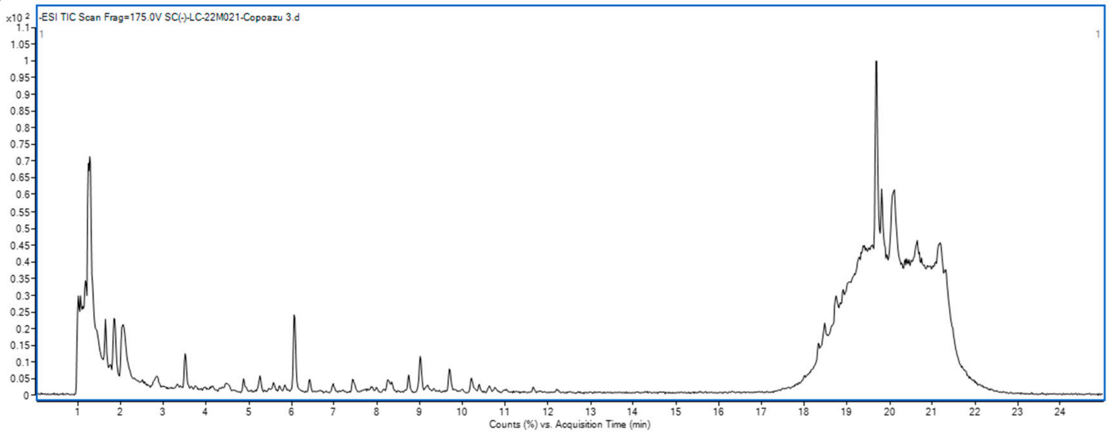

b)

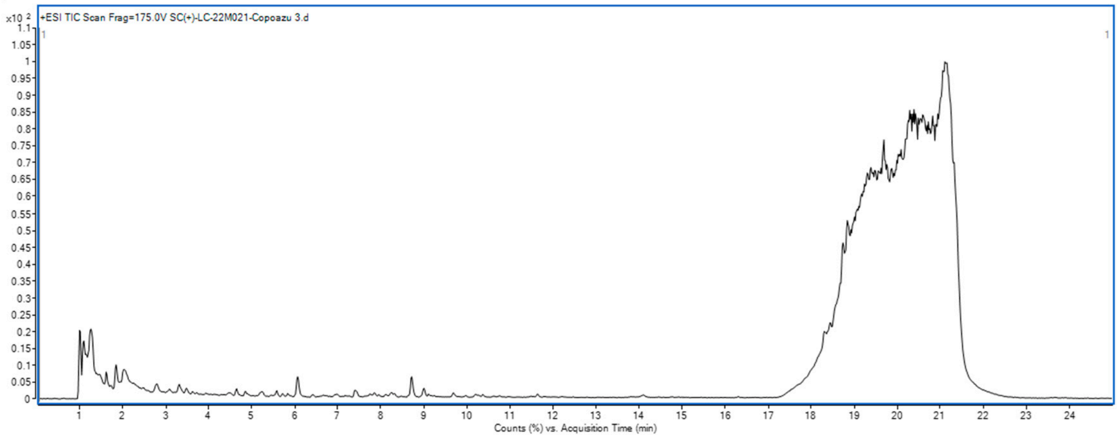

c)

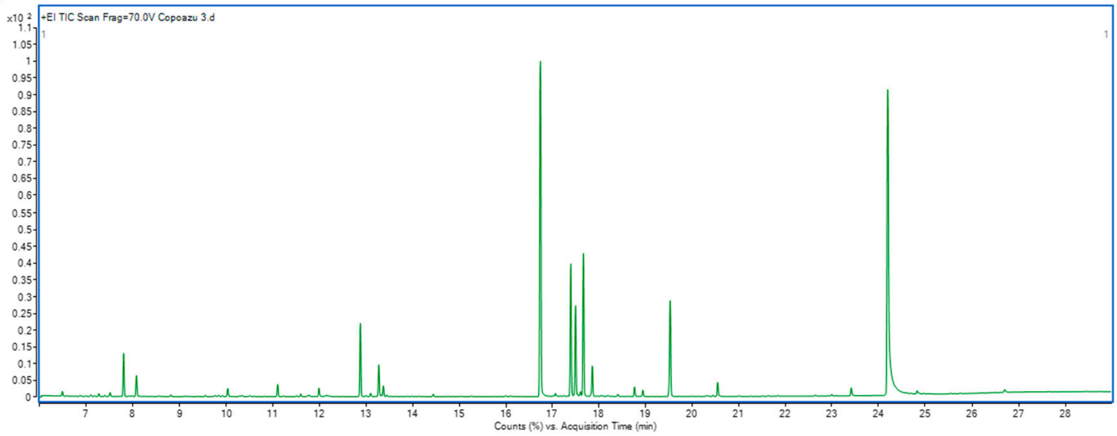

Figure S8. Chromatograms of the *Theobroma grandiflorum* ripe pulp extract obtained by **a)** LC-QTOF-MS(-), **b)** LC-QTOF-MS(+), and **c)** GC-QTOF-MS.

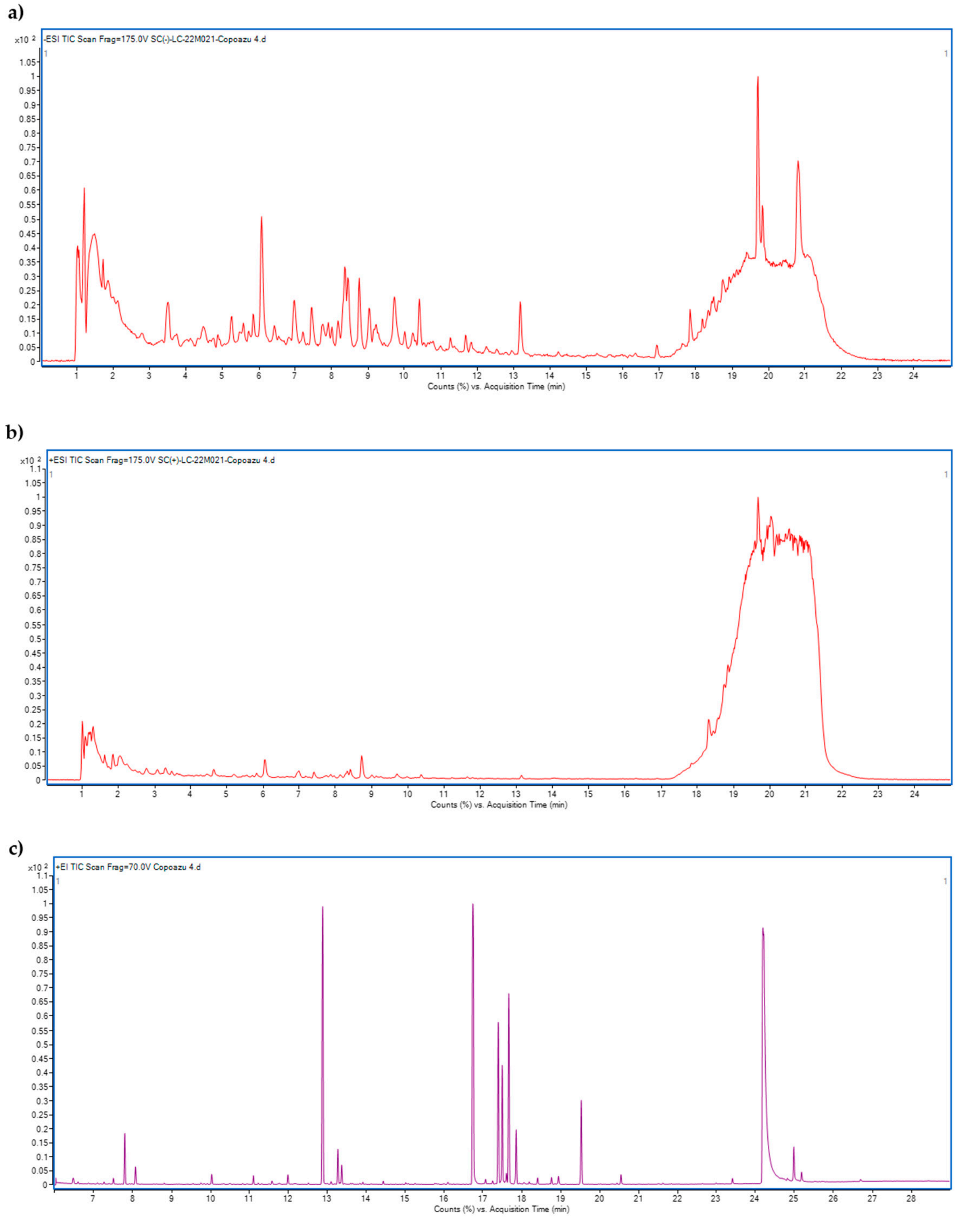

Figure S9. Chromatograms of the *Theobroma grandiflorum* unripe pulp extract obtained by **a)** LC-QTOF-MS(-), **b)** LC-QTOF-MS(+), and **c)** GC-QTOF-MS.

a)

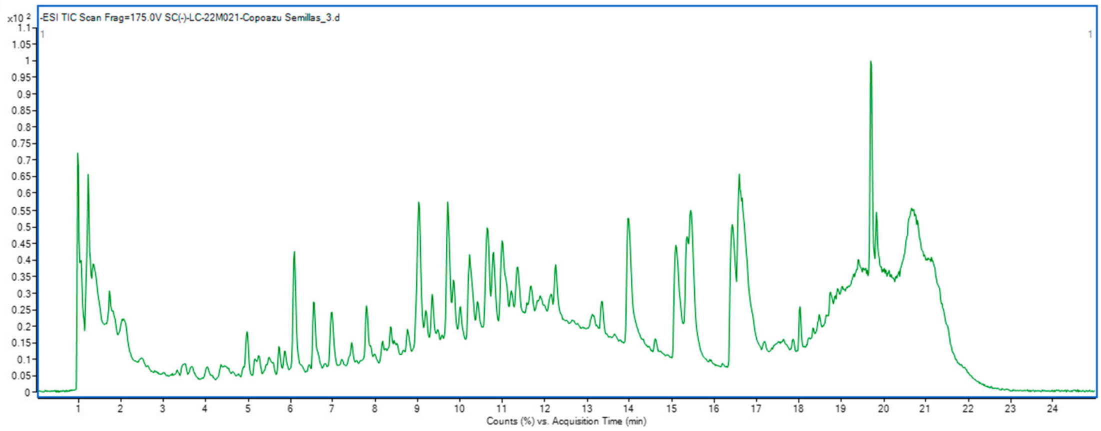

b)

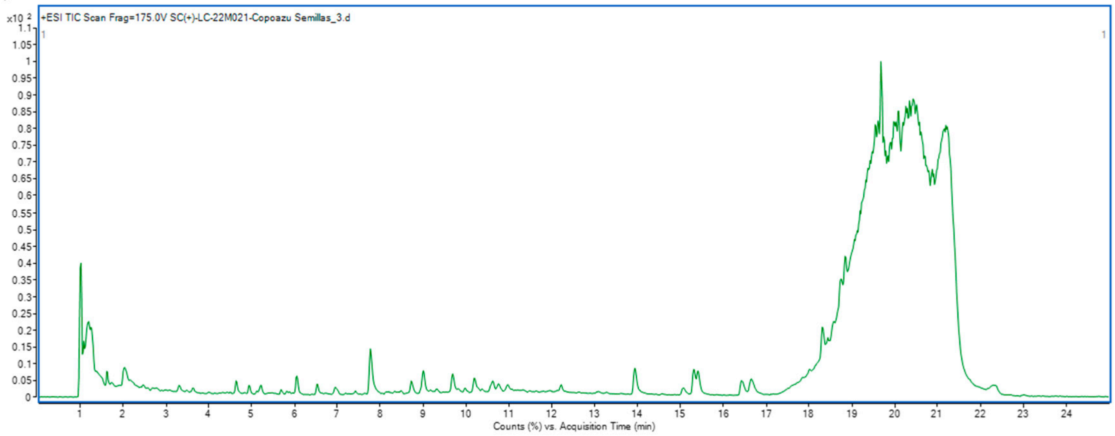

c)

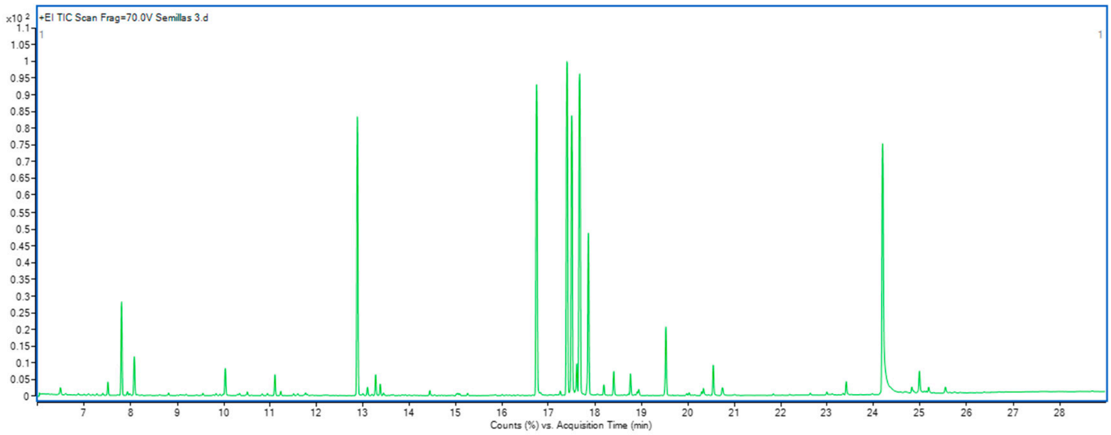

Figure S10. Chromatograms of the *Theobroma grandiflorum* ripe seed extract obtained by **a)** LC-QTOF-MS(-), **b)** LC-QTOF-MS(+), and **c)** GC-QTOF-MS.

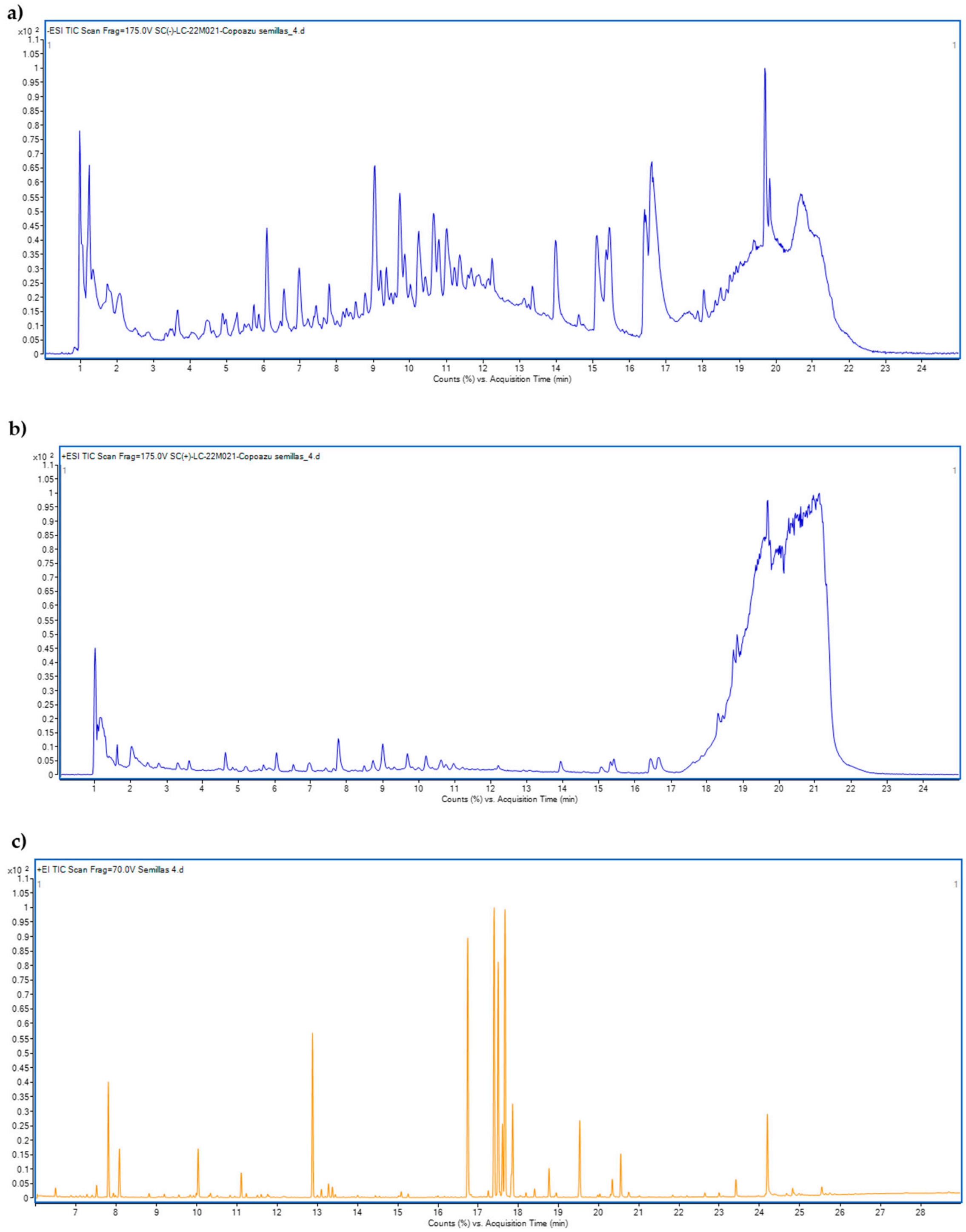

Figure S11. Chromatograms of the *Theobroma grandiflorum* unripe seed extract obtained by **a)** LC-QTOF-MS(-), **b)** LC-QTOF-MS(+), and **c)** GC-QTOF-MS.
